# The Making of Synaptic Ribbons: Carboxy-Terminal Tagging of RIBEYE B-Domain Reduces Steady-State Levels of RIBEYE and Its Efficacy to Build Synaptic Ribbons

**DOI:** 10.3390/cells15171583

**Published:** 2026-08-31

**Authors:** Ajay Kesharwani, Marie-Lisa Eich, Amrita Mukherjee, Stephan Maxeiner, Gabriele Kiefer, Gabriela Krasteva-Christ, Karin Schwarz, Frank Schmitz

**Affiliations:** Institute of Anatomy and Cell Biology, Medical School, Saarland University, Kirrbergerstr. 100, 66421 Homburg, Germany; ajay.kesharwani1@uni-saarland.de (A.K.); marie-lisa.eich@charite.de (M.-L.E.); mukherjee.amrita85@gmail.com (A.M.); stephan.maxeiner@uni-saarland.de (S.M.); gabikiefer@gmx.net (G.K.); gabriela.krasteva-christ@uks.eu (G.K.-C.); karin.schwarz@uni-saarland.de (K.S.)

**Keywords:** ribbon synapse, synaptic ribbon, RIBEYE, CtBP2, retina, RIBEYE A-domain, RIBEYE B-domain

## Abstract

RIBEYE is the central building block of synaptic ribbons, the eponymous presynaptic specializations of ribbon synapses. RIBEYE consists of two major protein regions: a unique amino-terminal, proline-rich A-domain and a carboxy-terminal, NADH-binding B-domain, which is largely identical to tetramer-forming nuclear protein CtBP2. In RIBEYE knockout mice, synaptic ribbons are completely absent, emphasizing the central role of RIBEYE in making synaptic ribbons. In the present study, we used a previously generated transgenic RIBEYE mouse line, in which a large protein tag was fused to the carboxy-terminus of RIBEYE-B-domain (RIBEYE-C-TG). We generated RIBEYE-C-TG-expressing RIBEYE knockout mice to study the impact of carboxy-terminal tagging of RIBEYE B-domain on synaptic ribbon formation. We applied high-resolution confocal immunofluorescence microscopy, conventional and E-PTA transmission electron microscopy to analyze whether carboxy-terminally tagged RIBEYE-C-TG rescues synaptic ribbon deficiency in RIBEYE knockout mice and restores synaptic ribbon in situ. High-resolution immunofluorescence microscopy revealed that transgenic RIBEYE-C-TG expression rescued synaptic ribbon formation in RIBEYE knockout mice. Electron microscopy confirmed that transgenic ribbons consisting of transgenic RIBEYE-C-TG were made, anchored to the active zone, and associated with synaptic vesicles. But transgenic synaptic ribbons formed by RIBEYE-C-TG were considerably smaller in size than synaptic ribbons in control mice. The expression level of RIBEYE-C-TG protein was much lower than untagged RIBEYE protein as demonstrated by qualitative and quantitative Western blot analyses. The decreased expression level of RIBEYE-C-TG protein is not due to decreased activity of the transgenic RIBEYE promoter or alterations of the 5′-UTR as shown by RT-qPCR analyses of RIBEYE transcripts and reporter assays. Our findings suggest that carboxy-terminally tagged RIBEYE-C-TG protein is less stable and less efficient in making synaptic ribbons than the untagged RIBEYE protein. Based on the findings in this study and on recently published structural data from other groups on RIBEYE and RIBEYE B-domain/CtBP2, we predict that carboxy-terminal tagging of RIBEYE B-domain, placed at the interaction interface of RIBEYE B-domain tetramers/RIBEYE B-domain filaments, could interfere with the formation and/or stability of RIBEYE B-domain-dependent oligomeric complexes which are likely essential to build the synaptic ribbon.

## 1. Introduction

Ribbon synapses are specialized chemical synapses in the retina, inner ear, and pineal gland [1,2]. They are capable of fast and continuous synaptic transmission and coordinated multi-vesicular exocytosis [1,2]. Ribbon synapses contain structural and functional specializations to perform their special role. The most conspicuous morphological specialization is the eponymous synaptic ribbon. The synaptic ribbon is an electron-dense structure associated with the active zone neurotransmitter release site and binds many vesicles in dense, spatially regular patterns, as shown, e.g., by EM tomography [3,4] or freeze-fracture analyses [5]. The size and shape of the synaptic ribbon are important for synaptic vesicle binding and for the physiological performance of the synapse [2,6].

In the retina, glutamatergic ribbon synapses are formed by photoreceptors and retinal bipolar cells [1,2]. The mouse retina is a rod photoreceptor-dominated retina, with more than 95% of the photoreceptors being rod photoreceptors [1,2,7]. Consequently, the outer plexiform layer of the retina contains mostly rod synapses. Rod photoreceptor synapses form a morphologically homogeneous synapse population with well-characterized ultrastructural features and are particularly suitable for morphological analyses. Rod photoreceptor synapses contain a single, particularly large synaptic ribbon that typically appears bar-shaped in transmission electron microscopy (TEM), if cross-sectioned [8,9,10].

The RIBEYE protein is the main unique building block of synaptic ribbons [11,12]. RIBEYE consists of a unique proline-rich amino-terminal A-domain and a carboxy-terminal B-domain that is largely identical to the transcriptional regulator protein CtBP2 [11,13]. Only the first 20 amino-terminal amino acids of CtBP2, encoded by a CtBP2-specific 5′ exon, are lacking in RIBEYE. Instead of these CtBP2-specific 20 amino acids (aa), RIBEYE contains the 563 aa long RIBEYE A-domain, which is encoded by a single 5′ exon in the *RIBEYE*/*CtBP2* gene. The shared carboxy-terminal sequences of RIBEYE and CtBP2 are encoded by 8 common 3′ exons which are spliced to the corresponding protein-specific 5′ exon. Thus, the RIBEYE/CtBP2 gene is a bifunctional gene that generates two different gene products driven by two different promoters and subsequent alternative splicing: CtBP2, which is ubiquitously expressed, and RIBEYE, which is only expressed in ribbon synapses [11,14]. Interestingly, a splice variant of CtBP2, CtBP2-S, has also been identified that lacks the first 20 amino acids of CtBP2 giving rise to a CtBP2 protein (CtBP2-S) that is completely identical to RIBEYE B-domain [15]. RIBEYE B-domain/CtBP2 binds NAD(H) in a redox-sensitive manner with high affinity [16]. Recently, the oligomeric structure of CtBP2 has been solved [17,18,19,20,21]. CtBP2 was shown to form tetramers in an NAD(H)-dependent manner [18,19,20,21]. The protein sequence of RIBEYE A-domain is partly relatively divergent between species [11]. In contrast, RIBEYE B-domain protein sequence is highly conserved between species. RIBEYE B-domain binds NAD(H) and evolved from a family of D-isomer-specific 2-hydroxy acid dehydrogenases [11]. It consists of an NAD(H)-binding central sub-domain (NBD), a bipartite substrate-binding sub-domain (SBD), and an intrinsically unstructured Carboxy-terminus [22,23,24].

Synaptic ribbons are formed by neither CtBP2 nor CtBP2-S alone [15], emphasizing the essential role of RIBEYE A-domain in synaptic ribbon formation. On the other hand, RIBEYE B-domain is clearly also required for synaptic ribbon formation, as shown by RIBEYE knockin (RIBEYE KI) mice [25]. In RIBEYE KI mice, the replacement of RIBEYE B-domain by an unrelated protein [12] leads to a similar phenotype to deletion of RIBEYE A-domain, i.e., the complete and selective loss of synaptic ribbons in the retina and inner ear [25]. The reason for the essential role of RIBEYE B-domain in synaptic ribbon formation is only incompletely understood (see discussion). In the current study, we aimed to analyze whether carboxy-terminal tagging of RIBEYE B-domain influences the ability of RIBEYE to generate synaptic ribbons. We focused on rod photoreceptor ribbon synapses because these retinal synapses have a uniform morphology and possess impressively large synaptic ribbons that are particularly well suited to morphological analyses.

## 2. Materials and Methods

### 2.1. Mice

Mice were kept under a 12 h light/12 h dark cycle, standard laboratory animal housing conditions (≈20–22°C, ≈55% humidity), and were provided with standard food and water ad libitum. Animals were kept in an enriched surrounding. Animal health and welfare was monitored by the animal welfare officer of the university and independent veterinarians. All animal procedures were reviewed and approved by the local animal authorities and performed under the animal license GB 3-2.4.2.2-25-2020, issued on 29 August 2023. RIBEYE knockout (KO) mice and RIBEYE-C-TG transgenic mice (abbreviated as RIBEYE-C-TG mice) were previously generated and characterized [12,26,27]. The RIBEYE-C-TG transgenic mouse line was maintained on the genetic background of C57BL/6J wild-type (WT) mice for more than 30 generations and thus expressed endogenous mouse RIBEYE WT protein together with the RIBEYE-C-TG protein in previous studies [26,27]. Of note, for the generation of the RIBEYE-C-TG mice, cDNA encoding rat RIBEYE was used. Rat RIBEYE protein is very similar to mouse RIBEYE; however, few amino acid differences exist particularly in RIBEYE A-domain which is less strongly conserved than RIBEYE B-domain [11]. The overall similarity is about 97.3% between mouse and rat RIBEYE at the protein level. For Supplemental Figure S2 also rat retinas were analyzed. Rat eyes were obtained from the local animal facility and processed as described below within 10 min post-mortem.

In the present study, we crossed the two mentioned mouse lines, i.e., RIBEYE-C-TG mice and RIBEYE KO mice, with each other to generate homozygous RIBEYE KO mice that express carboxy-terminally tagged RIBEYE-C-TG protein only but no endogenous RIBEYE. Our final aim was to cross the RIBEYE-C-TG transgene into the RIBEYE knockout mouse line to obtain RIBEYE-C-TG expression on a pure homozygous RIBEYE knockout background (KO/KO). For this purpose, homozygous RIBEYE KO mice were bred with RIBEYE-C-TG mice to obtain RIBEYE-C-TG mice on the RIBEYE KO genomic background. During the generation of these mice, also other “intermediate” genotypes (i.e., KO/WT; KO/KO, KO/WT/+RIBEYE-C-TG, KO/KO/+RIBEYE-C-TG) of both sexes were obtained and analyzed.

### 2.2. Mouse Genotyping

Genomic DNA was isolated from surplus tissue pieces obtained during mouse ear tagging, and genotyping was performed as previously described [12,25,26,27]. RIBEYE WT reaction was performed with WT forward primer CTGCGGAAACCTGGAGCA (in the RIBEYE A-domain exon) and WT reverse primer: GACAAGACAGACACTATCT (in the first intron downstream of the RIBEYE A-domain coding exon), generating a 262 bp PCR product in case of a positive WT reaction. The KO reaction was performed with forward primer 11611: CTTGTGGCTGTGTACAGTTAGCT (located in the mouse RIBEYE promoter) and reverse primer 11612: GCCATCCAACCGAACTAGAGAA (located in the intron downstream of the RIBEYE A-domain encoding exon), resulting in a 412 bp amplicon for the knockout allele. RIBEYE-C-TG reaction was performed with forward primer 1 (F1) GCTTCGAATTCGGCACGAGGA located in the 5′-UTR; reverse primer R1 (R1): CCTGGTGCCCCTGGATGGGT (located in RIBEYE A-domain) resulting in a 396 bp amplicon; forward primer 2 (F2): CCACGGAGATCCGCCGAGCA (located in RIBEYE B-domain) and reverse primer 2 (R2): CGCCCTCGGATGTGCACTTGA (located in the tRFP encoding DNA), resulting in a 447 bp amplicon; forward oligo F3: GCTCGCCGTGAAAGAGTGGC (present in DNA encoding SNAP-tag) and reverse primer 3 (R3): GACGATGGGCGCGGAGCATA located in the 3′ UTR, resulting in a 578 bp amplicon.

### 2.3. Antibodies

#### 2.3.1. Primary Antibodies

Primary antibodies are summarized in Table 1.

#### 2.3.2. Secondary Antibodies

Secondary antibodies are summarized in Table 2.

### 2.4. Embedding of Retina Samples in Epoxy Resin for Immunohistochemistry

Embedding of retina samples into epoxy resin was performed as previously described [28,35,40]. This immunolabeling procedure is based on an aldehyde-free embedding method providing high signal-to-noise ratios and enabling high-resolution analyses through the preparation and immunolabeling of very thin sections. In brief, small retina samples were flash-frozen by liquid nitrogen-cooled isopentane. Next, samples were continuously cooled with liquid nitrogen and lyophilized with a vacuum of ≈10^−4^–10^−5^ Pa. During lyophilization, samples were kept under liquid nitrogen for about 2 days to ensure complete freeze-drying before the samples were gradually equilibrated to room temperature (RT). After equilibration to RT, samples were transferred to environmental pressure and infiltrated with freshly made epoxy resin [28,35,40]. To promote infiltration of the samples by epoxy resin, samples were maintained at 28 °C for ≈12 h on an overhead rotator. Afterward, infiltration was continued at room temperature for ≈24 h with continuous mild agitation. Epoxy-infiltrated retina samples were transferred to silicone embedding molds and polymerized for ≈48 h at 60 °C.

### 2.5. Immunolabeling of Epoxy Resin Sections of the Mouse Retina

Immunolabeling was performed with 0.5 μm-thin (“semi-thin”) epoxy resin sections, as previously described [28,35,40]. Semi-thin sections were cut with a Reichert UCT ultramicrotome (Reichert-Jung, Heidelberg/Nußloch, Germany) with a diamond knife (Histo-diamond; Diatome, Nidau, Switzerland) and collected on a drop of water placed on glass coverslips. Coverslips were heated for 15 min to allow firm attachment of the semi-thin section to the surface of the glass coverslip. Processing of the semi-thin resin sections for immunolabeling was performed as previously described [28,35,40]. For epoxy resin removal, sections were sequentially incubated with sodium methanolate (30% solution in methanol) for 10 min, a 1:1 mixture of xylol/methanol for 10 min, acetone (2×) for 10 min, water for 10 min, and PBS for 10 min. Afterward, the sections were incubated with the primary antibodies (summarized in Table 1 with the corresponding dilutions) at 4 °C overnight (ON) and, after several washing steps with PBS to remove unbound primary antibody, further incubated with the indicated secondary antibodies (summarized in Table 2) in a 1:1000 dilution for 1 h at room temperature. Immunolabeled sections were embedded in N-propyl gallate (NPG) antifade, as previously described [28,40].

### 2.6. Immunolabeling of Cryostat Sections from the Mouse (and Rat) Retina

Cryostat sections from the mouse retina were made from freshly isolated mouse eyes, largely as previously described [11,41]. Posterior eyecups were isolated as described above within 5 min post-mortem and flash-frozen in liquid nitrogen-cooled isopentane. Cryostat sections were made with a Leica cryostat CM1950 (Leica Biosystems, Wetzlar, Germany). Cryostat sections were air-dried for 30 min at RT and processed for direct endogenous fluorescence (i.e., tRFP fluorescence) and/or for immunohistochemistry, as described above for immunolabeling of semi-thin epoxy resin sections. These “native” cryostat sections are shown in Supplemental Figure S1. For the experiments shown in Supplemental Figure S2, cryostat sections of mouse and rat retinas were also used. For these experiments, cryostat sections were collected on glass coverslides (as described above) but were put on a heating pad (60 °C) for ≈30 min to promote adherence of the cryostat sections to the glass surface and to promote protein unfolding (protein denaturation). Immunolabeling was performed as described above for semi-thin epoxy resin sections. Primary and secondary antibodies were used as described in Table 1 and Table 2 and as described in the respective experiments. Negative controls were done by omitting the primary antibody with all other steps remaining the same.

### 2.7. Confocal Microscopy and Colocalization Analysis

Confocal microscopy was performed with an A1R confocal microscope (A1R, Nikon, Düsseldorf, Germany), as previously described [12,28,35,40,42,43]. Image acquisition was controlled with the NIS Elements software AR3.2, 64 bit (Nikon, Düsseldorf, Germany), and confocal images were acquired with a 60× Plan-Apochromat oil immersion objective (NA 1.4) (Nikon, Düsseldorf, Germany). Colocalization analysis was performed using Fiji (ImageJ, NIH, version 1.54P) [44,45] with the JACoP plugin (version 2.1.4) [46].

### 2.8. Transmission Electron Microscopy

For the ultrastructural analyses, homozygous RIBEYE knockout (KO/KO) mice, heterozygous RIBEYE knockout (KO/WT) mice, homozygous RIBEYE knockout (KO/KO) mice expressing transgenic RIBEYE-C-TG (KO/KO/+RIBEYE-C-TG), and heterozygous RIBEYE knockout (KO/WT) mice expressing RIBEYE-C-TG (KO/WT/+RIBEYE-C-TG) of both sexes were analyzed by transmission electron microscopy (TEM). Tissue samples were processed for TEM largely as previously described [12,25,40]. Mouse eyes were enucleated within 5 min post-mortem and hemisected coronally to remove the anterior eyecup. The posterior eyecups with the retina were fixed with 2% (wt/vol) freshly depolymerized paraformaldehyde (in PBS, pH 7.4) and 2.5% (vol/vol) glutaraldehyde (in PBS) for ≈12 h (at 4 °C with constant gentle agitation). Following several washes with PBS and then with 100 mM cacodylate buffer (pH 7.4), retina samples were postfixed with 1% (wt/vol) OsO_4_, 1.5% (wt/vol) K_4_[Fe(CN)_6_] × 3H_2_O in 100 mM cacodylate buffer, pH 7.4 for 1 h (at 4 °C on rotating shaker). After three washes with 100 mM cacodylate buffer, pH 7.4, and H_2_O, samples were equilibrated with 50 mM Na-maleate in H_2_O (pH 5.0). Next, samples were contrasted with 2% (wt/vol) uranyl acetate (UA) in 50 mM Na-maleate (pH 5.0) for 3 h (at 4 °C on a rotary shaker in the dark). Samples were washed with maleate buffer, H_2_O, and then dehydrated with an ascending, graded series of ethanol (30%, 50%, 70%, 80%, 90%, 99%) and pure propylene oxide (15 min at each step at RT). Propylene oxide was gradually replaced by a mixture of propylene oxide and increasing volumes of epoxy resin (3/1, 1/1, 1/3 (vol/vol); 1 h each at RT). Finally, the samples were infiltrated with pure epoxy resin overnight (ON) at RT with mild agitation and transferred to silicone-embedding molds for polymerization. Polymerization of the epoxy resin-infiltrated samples was performed at ≈60 °C for ≈48 h. Ultrathin sections (≈70 nm in thickness) were cut with a diamond knife (Diatome, Nidau, Switzerland) and a Reichert UCT ultramicrotome (Reichert-Jung, Heidelberg/Nußloch, Germany). For transmission electron microscopy (TEM), ultrathin sections were analyzed with a Tecnai Biotwin12 digital transmission electron microscope (FEI) equipped with a Megaview III digital camera (Gatan) and controlled by iTEM acquisition software 5.0 (Olympus, Hamburg, Germany). The transmission electron microscope was operated at an accelerating voltage of 100 kV.

In the present study, we focused on rod photoreceptor ribbon synapses, which are the dominant photoreceptor synapses in the mouse retina [2,7], and rod photoreceptor synapses in the outer plexiform layer (OPL) can be safely identified by their localization and typical ultrastructural appearance by transmission electron microscopy [2,6,47]. Rod photoreceptors possess a single large active zone associated with a single, particularly large synaptic ribbon [2,47]. Cone synapses are larger in size than rod synapses and more heterogeneous. They possess multiple active zones with smaller synaptic ribbons [2,47].

### 2.9. Quantification of Synaptic Ribbon Height by Transmission Electron Microscopy

Quantification of synaptic ribbon height was performed as previously described [40]. For standardization, synaptic ribbon height was only analyzed in cross-sections of the photoreceptor synapse, i.e., synapse sections in which the presynaptic active zone and the postsynaptic dendrites were clearly visible. For measurement of synaptic ribbon height, only rod photoreceptor synapses were analyzed in which the active zone could be clearly visualized and in which the typical postsynaptic configuration consisting of horizontal and bipolar cells was visible (postsynaptic triad/tetrad). In this way, only synaptic ribbons that were cut perpendicular to the active zone were considered for the analyses. For the analyses of ribbon height in rod photoreceptor synapses, electron micrographs of rod photoreceptor ribbon synapses were typically captured at magnifications of 20,000×–44,000×. Synaptic ribbon height was measured using the FEI TEM Imaging & Analysis (TIA) software (version 4.15).

### 2.10. Staining of Retina Samples with Ethanolic Phosphotungstic Acid (E-PTA) for Transmission Electron Microscopy

Ethanolic phosphotungstic acid (E-PTA) staining for transmission electron microscopy (TEM) enables a relatively selective visualization of synaptic ribbons and pre- and postsynaptic densities in the retina [25,48,49]. E-PTA staining for TEM was performed, largely as previously described [25,48,49]. For E-PTA electron microscopy, 4–7-month-old mice of the genotypes described in the respective experiments (both sexes) were analyzed. Posterior eyecups were dissected from the isolated eyes within 5 min post-mortem. The posterior eyecups with the attached retina were fixed using a two-step procedure. Samples were first immersed in 4% paraformaldehyde (PFA) in PBS (pH 7.4) fixation solution (ON at 4 °C with mild agitation), followed by a second fixation step with 2.5% glutaraldehyde in PBS (ON, at 4 °C with mild agitation). Following several washes with PBS, samples were dehydrated with an ascending ethanol concentration series (30%, 50%, 70%, 80%, 90%, 99%), 15 min each step at RT. Next, samples were incubated for 10 min in absolute ethanol (100%) before samples were stained for 1.5 h with 1% phosphotungstic acid (wt/vol) in absolute ethanol to which five drops of 95% absolute ethanol per 10 mL of staining solution were added [25,48,49]. Samples were stained with E-PTA solution for 1.5 h at RT on an orbital shaker. Then, E-PTA solution was removed and replaced by pre-cooled, ice-cold propylene oxide. Propylene oxide was kept ice-cold to prevent the development of an exothermic reaction [49,50]. After ≈10 min, propylene oxide was changed once, and samples were incubated in propylene oxide for 30 min. Next, tissue was infiltrated with epoxy resin. Epoxy resin was replaced once, and the samples were infiltrated with epoxy resin for ≈24 h with mild agitation. Epoxy resin was polymerized for ≈48 h at 60 °C. Ultrathin sections were cut from the polymerized sample blocks with a diamond knife and mounted on 100 mesh copper grids. On purpose, sections from E-PTA-stained samples were not contrasted with uranyl acetate or lead citrate. For analysis, sections were examined with a Tecnai Biotwin12 (FEI) transmission electron microscope, as described above.

### 2.11. Western Blot Analyses

For Western blot (WB) analyses, mice from different genotypic backgrounds were sacrificed by cervical dislocation after isoflurane inhalation. Eyes were enucleated and retinas were isolated. SDS PAGE and WB were performed as described previously [12]. The isolated retinas were lysed in RIPA lysis and extraction buffer (100 mM Tris, 150 mM NaCl, 1 mM EDTA, 1% Triton X-100, 0.5% NP40, 0.5% sodium deoxycholate). Retinas in RIPA lysis and extraction buffer were sonicated using an ultrasonic probe (Bandelin Sonoplus ultrasonic homogenizer; Berlin, Germany; 20% amplitude setting; three pulses of sonication). After sonication, the lysate was kept on ice for 20 min to allow lysis to be completed, followed by centrifugation of the lysate at 13,000 rpm in a tabletop centrifuge for 60 min at 4 °C. The supernatant was collected after centrifugation. Total protein concentration of the retinal lysate was quantified using Bradford colorimetry assay [51]. A serial dilution of known bovine serum albumin (BSA) concentration was used to develop a standard linear calibration curve. The concentration of the proteins in the retinal lysate was determined with the help of the standard curve. A total of 30 µg of protein from the retinal lysate (collected supernatant) was treated with modified Laemmli buffer (16% SDS (wt/vol), 20% glycerol (vol/vol), 1M Tris, 5% 2-βME (vol/vol), 0.02% (wt/vol) Bromo Phenol Blue dye) and heated for 10 min at 95 °C. The heated samples were separated on 8% acrylamide sodium dodecyl sulfate polyacrylamide gel electrophoresis (SDS-PAGE), if not denoted otherwise. Pre-stained protein ladder (#26616, Thermo-Scientific, Dreieich, Germany) was used as a molecular weight standard. Following electrophoresis, the proteins were transferred from the gel to a nitrocellulose membrane (Protran 0.45 µm, GE Healthcare, Düsseldorf, Germany) at 60 V for 6 h at 4 °C. After electrotransfer, the membrane was washed three times for five minutes each with 0.1% Tween in Tris-buffered saline (TBST) followed by blocking with 5% wt/vol skim milk in 0.1% TBST for 1 h at RT. The membrane was then washed and incubated with the indicated primary antibodies, i.e., either mouse monoclonal anti-RIBEYE B-domain (clone 2D9) or mouse monoclonal anti-actin (clone C4), in blocking buffer ON at 4 °C. After incubation with the indicated primary mouse antibody, the membrane was washed in 0.1% TBST and incubated with the chicken anti-mouse Alexa488 Fluor-conjugated secondary antibody for 2 h at RT (1:5000 dilution). Following 2 h of incubation with secondary antibody, the membrane was washed and imaged using the Bio-Rad ChemiDoc^TM^ MP imaging system (Bio-Rad Laboratories, Hercules, CA, USA) with Image Lab software (version 6.1). Please note that the Alexa 488 channel in the Bio-Rad ChemiDoc^TM^ MP imaging system (Bio-Rad Laboratories, Hercules, CA, USA) was used both for the detection of RIBEYE-C-TG transgenic protein (detected by the monoclonal RIBEYE 2D9 primary antibody) and for the detection of actin (by the monoclonal actin C4 antibody). First, we used mouse monoclonal antibody against the RIBEYE B-domain (clone 2D9) to probe the expression of RIBEYE-C-TG transgenic protein followed by a mouse monoclonal anti-actin antibody (clone C4) without stripping the membrane (Figure 11(A1,A2)). Actin (at ≈43 kDa) served as the loading control for the quantification of relative RIBEYE-C-TG transgenic protein expression in the indicated transgenic mice. Part of the WB data were also analyzed with a Bio-Rad ChemiDox XRS system (Bio-Rad Laboratories, Hercules, CA, USA) and enhanced chemiluminescence (ECL) detection, as indicated in the respective experiments.

In the WB experiments of the reporter protein expression assays (cf. Figure 13), proteins were first subjected to 10% SDS-PAGE and subsequently electrotransferred to a nitrocellulose membrane at 50 V, 10 h. The nitrocellulose membranes with the electrotransferred proteins were incubated with 10% milk powder in PBS (45 min at RT) to block nonspecific protein-binding sites before the membranes were incubated with a mixture of the two primary antibodies, i.e., rabbit polyclonal antibody against HA (Abcam) and mouse monoclonal antibody against actin (clone C4) ON, 4 °C (Table 1). After several washes with PBS to remove unbound primary antibody, membranes were incubated simultaneously with a mixture of the respective fluorophore-conjugated secondary antibodies (1 h at RT) (donkey anti-mouse Alexa488; donkey anti-rabbit Alexa568; Table 2). After subsequent washes with PBS, antibody binding was visualized by emission of the respective fluorescence signals in a Bio-Rad ChemiDoc^TM^ MP imaging system (Bio-Rad Laboratories, Hercules, CA, USA). Actin served as a loading and quantification reference in these experiments. Reporter protein signals (as obtained with the polyclonal HA antibody) were normalized to the signals obtained with the monoclonal actin antibody.

### 2.12. Quantification of Western Blot Bands

RIBEYE-C-TG expression in retinal lysates was measured by quantification of WB bands. For the quantification of the RIBEYE-C-TG transgenic protein expression by WB, the Bio-Rad ChemiDoc^TM^ MP imaging system (Bio-Rad Laboratories, Hercules, CA, USA) with Image Lab software (version 6.1) was used. For quantitative WB analyses, images were always acquired with the “Optimal Auto-Exposure” option of the Bio-Rad Chemidoc MP Imaging system to ensure accurate quantification with maximal linear range, high sensitivity and avoiding pixel saturation. Fluorescence WB images from the Bio-Rad Chemidoc MP Imaging system were exported as 16-bit raw TIFF image files using Image Lab software (version 6.1). The fluorescence integrated density of the RIBEYE-C-TG transgenic protein band, the RIBEYE WT protein band and the actin band in heterozygous (WT/KO) mice and RIBEYE knockout mice (KO/KO) were quantified by using Fiji software (ImageJ, NIH) with the macro plug-in: BandPeakQuantification for Western blot quantification (doi:10.17504/protocols.io.7vghn3w). Briefly, rectangular regions-of-interests (ROIs) were drawn around the protein band using the rectangular selections tool in ImageJ. The Band Peak Quantification Tool macro was run selecting median background subtraction with three pixel top/bottom regions to accurately account for the local background. The final values given by the tool were the background-corrected integrated density of the respective protein bands. The integrated density of the RIBEYE-C-TG transgenic protein band and/or the WT RIBEYE band in WT/KO mice was normalized to the integrated density of the actin signal in the same respective lane which served as the loading control protein. To compare the relative expression of transgenic RIBEYE-C-TG protein with the heterozygous (WT/KO) RIBEYE protein, the heterozygous (WT/KO) wild-type RIBEYE protein band integrated density was set to 100%.

Statistical analyses were performed using GraphPad Prism software version 10.6.1. The Shapiro–Wilk test was performed to check whether the data from the individual genotypes were normally distributed. Brown–Forsythe and Welch ANOVA tests (assuming unequal standard deviations/variances for the different genotypes) were applied for multiple comparisons as the data sets were normally distributed.

### 2.13. Reverse Transcription-Quantitative PCR (RT-qPCR)

The retina of each mouse was collected in 1 mL TRIzol reagent (Invitrogen, Carlsbad, CA, USA), supplemented with ceramic beads and homogenized using a FastPrep-24 5G speed mill (MP Biomedicals, Irvine, CA, USA). The samples were centrifuged for 1 min at 13,000 rpm in a tabletop centrifuge and 900 µL of the supernatant was transferred to a new 2 mL tube. An equal amount of 100% ethanol was added to each sample and thoroughly mixed. Subsequently, total RNA was isolated from each sample using the Direct-zol Mini RNAPrep Plus kit (Zymo Research Europe GmbH, Freiburg, Germany) according to the manufacturer’s protocol. Samples were eluted from the column resin in a total volume of 120 µL nuclease-free water and nucleic acid concentration was quantified using a NanoDrop One photometer (VWR International GmbH, Bruchsal, Germany). To determine the potential contamination of the eluted RNA with genomic DNA, a total of 10 µg of RNA from all samples was treated with the TURBO DNA-free™ kit (Invitrogen). Successful treatment was assessed by RT-qPCR employing a β-actin (*Actb*)-specific probe-based hexachlorfluoresceine (HEX) dye-conjugated assay (Mm.PT39a.22214843.g, PrimeTime^TM^ predesigned qPCR Reference gene Assay for mouse, Integrated DNA Technologies, Coralville, IO, USA), which results in a 147 bp amplicon based on amplification of cDNA, or in a 272 bp amplicon resulting from contaminations with genomic DNA including the 125 nucleotide spanning intron. Upon PCR amplification, all samples were separated on a 1% agarose gel for 60 min to determine successful DNase treatment and genomic DNA removal (cf. Supplemental Figure S4).

To assess transcriptional activity of the RIBEYE promoter in RIBEYE deficient (KO/KO), heterozygous (KO/WT) and transgenic RIBEYE-C-TG mouse lines (RIBEYE-C-TG), DNA-free RNA from the respective retina isolates per genotype was loaded in triplicate, respectively, and submitted to RT-qPCR analysis. Each sample contained 5 µL Luna^®^ Universal Probe One-Step RT-qPCR 2x master mix (New England Biolabs GmbH, Frankfurt am Main, Germany), 2.5 µL nuclease-free water, 0.5 µL enzyme mix, 0.5 µL *Actb* assay (HEX-dye conjugated, see above), 0.5 µL RIBEYE assay (Fluorescein amidite (FAM) dye-conjugated), and 1 µL of RNA (100 ng/µL). The RIBEYE probe-based assay was purchased from Integrated DNA Technologies and comprised forward and reverse oligonucleotides annealing to the RIBEYE-specific exon coding for the RIBEYE A-domain AACCATGCTAACTCCAGAACC, and the subsequent exon common to RIBEYE and CtBP2, ATCACAGAAGGCCACAGTG, respectively. The nucleotide sequence of the probe is as follows: CCCCAGATCATGAACGGCCCC. RIBEYE probe assay was developed using IDT’s Real-Time PCR Tool (https://eu.idtdna.com/scitools/Applications/RealTimePCR/default.aspx, accessed on 27 April 2026) based on the mouse annotated RIBEYE/CtBP2 gene sequence (https://www.ncbi.nlm.nih.gov/; Gene ID: 13017, accessed on 27 April 2026). We would like to point out that for the generation of the transgenic mice, rat RIBEYE cDNA was used (Gene ID: 81717), which results in a single nucleotide polymorphism in the forward oligonucleotide at position 13 (a T-to-A mismatch). Assessment of assay efficacy comparing rat and mouse cDNA showed no difference in assay performance. PCR reactions were run on a CFX Connect Real-Time System (Bio-Rad Laboratories, Feldkirchen, Germany) with the following temperature profile: 55 °C, 10 min (reverse transcription); 95 °C, 1 min (initial denaturation); 95 °C, 10 s (denaturation), 60 °C, 30 s (extension + plate read), 40 cycles. Data were analyzed as RIBEYE transcription standardized to the internal reference gene *Actb* as has been reported previously [52]. Data analysis was performed, and statistical significance was tested utilizing Brown–Forsythe and Welch ANOVA with Dunnett T3 post hoc correction for multiple comparisons using GraphPad Prism software 10.6.1.

### 2.14. Cloning of a Eukaryotic Reporter Construct for Checking Translation Efficiency

Cloning of a reporter protein-encoding eukaryotic expression vector in a CMV-based promoter containing either the upstream 5′-UTRs of the RIBEYE gene or the 5′-UTR of the transgene was conducted using Gibson assembly [53]. For this purpose, pEGFP-N1 (Clontech) was linearized by restriction digest with *Xho*I and *Not*I. The insert was obtained as a synthetic DNA construct (gBlock, Integrated DNA Technologies (IDT), Coralville, IO, USA). The gBlock contained 21 nucleotides of overlapping vector sequences at both ends. At the 5′ end of the gBlock vector sequence was followed by the 5′-UTR of the RIBEYE gene (or the 5′-UTR of the transgene) with the corresponding identical Kozak sequence followed by the sequence encoding the reporter protein consisting of tRFP, SNAP-tag, and HA-tag, followed by a STOP codon. The 3′ end of the gBlock was completed by 21 nucleotides of vector sequence. Gibson assembly by homologous recombination was performed according to the manufacturer’s protocol using the Gibson assembly cloning kit (New England Biolabs). Insert-containing plasmids were identified by restriction digest with *Nhe*I and *Not*I. Plasmids were verified by sequencing.

### 2.15. HEK293 Cell Culture and Transfection with Plasmids

HEK293 cells were cultured, as previously described [11] using DMEM cell culture medium supplemented with 10% FCS (Gibco, Thermo Fisher Scientific, Waltham, MA, USA) for cell culture. HEK293 cells were transfected with a transfection kit (Perfectin^TM^; Genlantis/AMSBIO, 1822 BH Alkmaar, The Netherlands) with 2 μg of column-purified plasmid DNA, according to the manufacturer’s instructions. For transfections, HEK293 cells were cultured in 10 cm culture dishes and transfected at a cell density of ≈80%. Cells were harvested 2 days after transfection. Cells were washed with ice-cold PBS before being collected with a disposable cell scraper and harvested by centrifugation (≈500× *g*, 10 min at RT). Cell extracts were generated, processed for SDS-PAGE and WB as described above for the retina. After blocking non-specific protein binding sites of the nitrocellulose membranes with 10% skim milk powder dissolved in PBS (45 min at RT), nitrocellulose membranes were simultaneously incubated with rabbit polyclonal antibody against the HA-tag of the reporter protein and mouse monoclonal antibody against actin (ON, 4 °C, Table 1), followed by simultaneous secondary antibody incubation with donkey anti-rabbit Alexa568 antibody and donkey anti-mouse Alexa488 (1 h at RT; Table 2). After several washes with PBS, the nitrocellulose membrane was scanned with a Bio-Rad ChemiDoc^TM^ MP imaging system (Bio-Rad Laboratories, Hercules, CA, USA). The HA signal was normalized to the actin signal in the same lane. Actin served as protein loading reference.

### 2.16. Statistical Procedures

All statistical analyses were done with GraphPad 10.6.1. Data presentation as SuperPlots and statistical analyses were performed according to [54] using GraphPad Prism 10.6.1. In all quantification figures, the arithmetic means of the individual independent experiments, the global mean of all experiments (±S.E.M.) and all individual datapoints were plotted accordingly, as specified in the respective figures. The specific statistical tests applied for the respective indicated datasets were given in the figure legends. Shapiro–Wilk/Kolmogorov–Smirnov tests were used to determine whether data were normally or non-normally distributed. Based on the normality testing, the indicated statistical tests for normally or non-normally distributed data were applied as specified in the corresponding figure legends. A *p*-value < 0.05 was considered statistically significant.

## 3. Results

In the present study, we generated transgenic mice that express carboxy-terminally tagged RIBEYE-C-TG in homozygous RIBEYE knockout (KO/KO) mice. This was done to analyze the potency of carboxy-terminally tagged RIBEYE-C-TG to generate synaptic ribbons. RIBEYE-C-TG contains a large protein tag, consisting of a combination of tag-RFP (237 amino acids (aa), ≈27 kDa), SNAP-tag (180 aa, ≈20 kDa), and HA-tag (9 aa, ≈1 kDa), fused in frame to the C-terminus of RIBEYE B-domain. We call this novel transgenic mouse line RIBEYE KO/KO/+RIBEYE-C-TG (abbreviated as KO/KO/+RIBEYE-C-TG) in the following text.

For the generation of the RIBEYE KO/KO/+RIBEYE-C-TG mice, two previously generated and characterized mouse lines were crossed with each other, i.e., (1). RIBEYE KO mice (RIBEYE (KO/KO) [12]), in which the RIBEYE A-domain-encoding exon had been deleted, resulting in the complete absence of RIBEYE and retinal synaptic ribbons [12], and (2). RIBEYE-C-TG transgenic mice expressing a full-length rat RIBEYE protein tagged at the carboxy-terminus with tRFP-, SNAP-, and HA-tags [26,27]. Offspring analyses demonstrated that the RIBEYE-C-TG transgene is present in the RIBEYE-C-TG mouse line as a single copy, as demonstrated by offspring analyses of 47 different litters (Supplemental Figure S1A). Half of the offspring obtained from a transgene-positive parent animal crossed with a non-transgenic animal was transgene positive, suggesting the presence of only a single transgenic RIBEYE-C-TG genomic copy in the RIBEYE-C-TG mouse line.

After several breedings of transgenic RIBEYE-C-TG mice with RIBEYE KO mice, we obtained mice expressing RIBEYE-C-TG on the genomic background of homozygous RIBEYE KO mice, as verified at the protein level by the following immunofluorescence labeling with the indicated antibodies. Please note that we used 0.5 μm-thin (“semi-thin”) epoxy resin sections for the subsequent immunofluorescence labeling at the light-microscopical level, if not denoted otherwise. Thin resin sections enable a particularly high resolution [55], and the applied semi-thin method provides low background and strong signal-to-noise ratios [28,35,40,42,43]. Of note, the endogenous fluorescence of fluorescent tags (i.e., tRFP) is lost during the embedding of fully dehydrated retina samples in the apolar, hydrophobic epoxy resin cured at high temperature (Supplemental Figure S1C, panel C1) because hydrophobic epoxy resins lead to a destruction/disturbance of the three-dimensional protein structure [56,57,58,59,60]. Only in native tissue [26,27] or in native cryostat sections (Supplemental Figure S1C, panel A1 and B1) is the direct endogenous fluorescence of tRFP preserved and directly visible without antibody immunolabeling.

**Figure 1:**
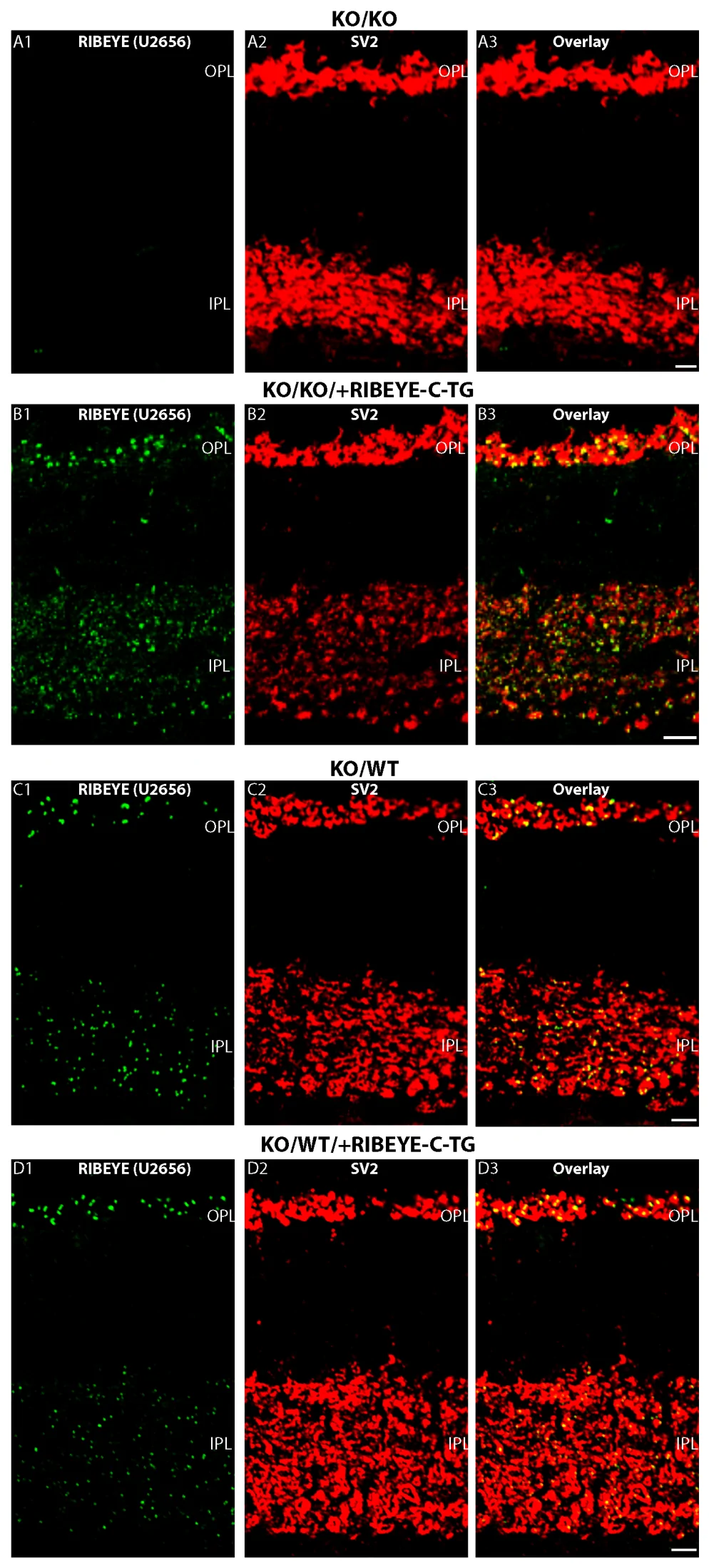
Transgenic expression of RIBEYE-C-TG rescues synaptic ribbon deficiency of RIBEYE KO mice as judged by immunofluorescence microscopy (confocal microscopy). 0.5 μm-thin (semi-thin) retina epoxy sections obtained from mice of the indicated genotypes were immunolabeled with rabbit polyclonal antibody against RIBEYE (U2656; [11]) to label synaptic ribbons and mouse monoclonal antibody against synaptic vesicle protein 2 (SV2; [29]) to label synaptic vesicles in presynaptic terminals of the OPL and the IPL. Representative immunofluorescence images are displayed. In (**A1**,**B1**,**C1**,**D1**), immunosignals obtained with the rabbit polyclonal RIBEYE B-domain antibody U2656 (green channel) are shown; in (**A2**,**B2**,**C2**,**D2**) immunosignals obtained with the mouse monoclonal SV2 antibody (red channel). Signals from the green channels (**A1**,**B1**,**C1**,**D1**) and the red channels (**A2**,**B2**,**C2**,**D2**) were merged in (**A3**,**B3**,**C3**,**D3**). Abbreviations: OPL, outer plexiform layer; IPL, inner plexiform layer. Scale bars: 5 μm.

In the retina semi-thin epoxy sections shown in Figure 1, presynaptic terminals were immunolabeled with antibodies against the synaptic vesicle protein SV2 [29], and synaptic ribbons were visualized with antibodies against RIBEYE, the main component of synaptic ribbons [11]. Immunolabeling of semi-thin retina resin sections of homozygous RIBEYE knockout mice (KO/KO) with rabbit polyclonal antibodies against RIBEYE (U2656; [11]) revealed the complete absence of synaptic ribbon-like RIBEYE signals in both synaptic layers of the retina, the outer plexiform layer (OPL) and the inner plexiform layer (IPL) of the retina (Figure 1(A1)), as previously observed [12,25,61]. In contrast, synaptic ribbons were formed in RIBEYE-C-TG-expressing RIBEYE KO mice (KO/KO/+RIBEYE-C-TG), as judged by immunolabeling of retinal sections with the rabbit polyclonal RIBEYE antibody U2656 [11] (Figure 1(B1–B3)). Both synaptic layers of the retina, the OPL and the IPL, contained RIBEYE-positive puncta. Thus, transgenic expression of RIBEYE-C-TG indeed appeared to rescue synaptic ribbon deficiency in RIBEYE knockout (KO/KO) mice, as judged by the presence of synaptic ribbon-typical RIBEYE signals in the OPL and IPL. In both genotypes, the SV2 immunosignals appeared very similar and were not affected by the different genotypes, emphasizing the specificity of the different RIBEYE immunosignals. As expected, synaptic ribbons were also present in a characteristic manner in the OPL and IPL of heterozygous knockout (KO/WT) mice, which also contained a wild-type allele (Figure 1(C1)), and in heterozygous RIBEYE knockout mice that additionally express transgenic RIBEYE-C-TG (KO/WT/+RIBEYE-C-TG) (Figure 1(D1)). In all genotypes the SV2 immunosignals were unaffected by the genetic manipulation, and the SV2 immunosignals allowed us to clearly identify the synaptic layers of the retina, the outer plexiform layer (OPL) and the inner plexiform layer (IPL).

**Figure 2:**
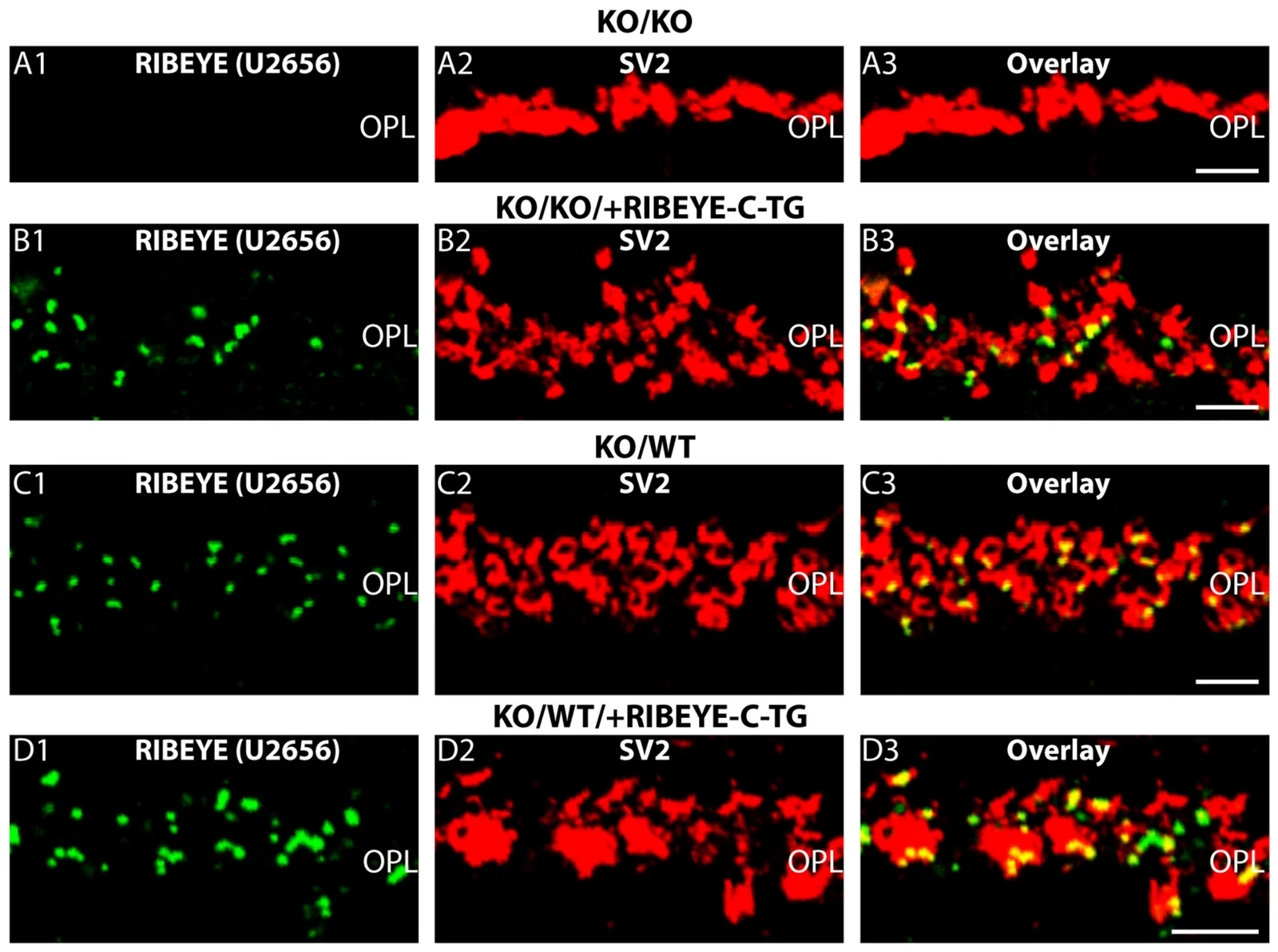
Transgenic expression of RIBEYE-C-TG rescues synaptic ribbon deficiency in photoreceptor synapses of RIBEYE KO mice. Semi-thin retina section obtained from mice of the indicated genotypes were immunolabeled as described above in Figure 1 with rabbit polyclonal antibody against RIBEYE (U2656; [11]) to label synaptic ribbons and mouse monoclonal antibody against synaptic vesicle protein 2 (SV2; [29]) to label synaptic vesicles in presynaptic terminals of the OPL at high magnification. Representative images are displayed. (**A1**,**B1**,**C1**,**D1**) shows immunosignals obtained with the rabbit polyclonal RIBEYE B-domain antibody U2656 (green channel); (**A2**,**B2**,**C2**,**D2**) immunosignals obtained with the mouse monoclonal SV2 antibody (red channel). Signals from the green channels (**A1**,**B1**,**C1**,**D1**) and the red channels (**A2**,**B2**,**C2**,**D2**) were merged in (**A3**,**B3**,**C3**,**D3**). Synaptic ribbons are absent in the OPL of RIBEYE KO mice (**A1**–**A3**), as also previously demonstrated [12]. Ribbons are re-formed in RIBEYE KO mice that also express transgenic RIBEYE-C-TG (**B1**–**B3**), as shown by confocal microscopy. Synaptic ribbons are also present in photoreceptors of KO/WT mice (**C1**–**C3**) and KO/WT mice that also express RIBEYE-C-TG (**D1**–**D3**). Abbreviations: OPL, outer plexiform layer. Scale bars: 5 μm.

Figure 2 presents higher magnification micrographs of the immunolabeled outer plexiform layer (OPL) in which the photoreceptor synapses are localized. Rod photoreceptor synapses, the predominant type of synapses in the OPL, have particularly large synaptic ribbons [1,2,7]. Synaptic ribbons were completely absent in the OPL of homozygous RIBEYE knockout (KO/KO) mice (Figure 2(A1)). The higher magnification images provided in Figure 2 confirmed that synaptic ribbons are made in photoreceptor synapses of RIBEYE-C-TG-expressing RIBEYE knockout mice (KO/KO/+RIBEYE-C-TG) (Figure 2(B1)), indicating that RIBEYE-C-TG can rescue synaptic ribbon formation.

Synaptic ribbons were also visible in photoreceptor synapses of heterozygous KO/WT mice (Figure 2(C1)) and RIBEYE-C-TG-expressing heterozygous (KO/WT/+RIBEYE-C-TG) mice (Figure 2(D1)).

**Figure 3:**
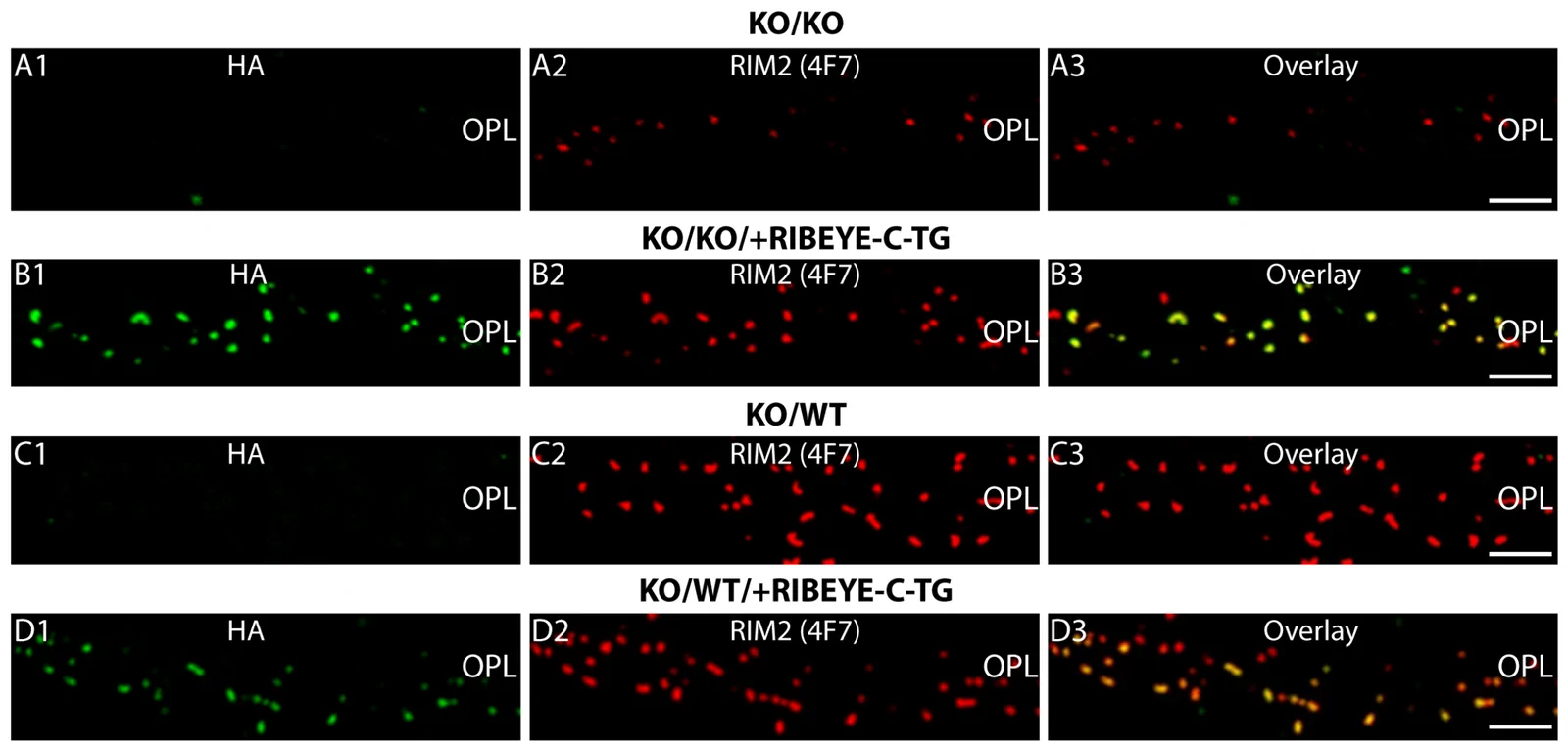
Synaptic ribbons made from transgenic RIBEYE-C-TG are properly localized to the presynaptic active zone neurotransmitter release sites. Representative images from semi-thin epoxy sections of mouse retinas from the indicated genotypes double-immunolabeled with mouse monoclonal antibodies against the active zone protein RIM2 (clone 4F7; [35]) (**A2**,**B2**,**C2**,**D2**) and rabbit polyclonal antibody against the HA-tag (**A1**,**B1**,**C1**,**D1**). The HA-tag antibody labels the transgenic RIBEYE-C-TG protein. Signals from the green channels (**A1**,**B1**,**C1**,**D1**) and the red channels (**A2**,**B2**,**C2**,**D2**) were merged in (**A3**,**B3**,**C3**,**D3**). The immunosignals obtained with the rabbit polyclonal antibody against the HA-tag and with the mouse monoclonal RIM2 antibody clone 4F7 showed a high degree of colocalization in KO/KO/+RIBEYE-C-TG mice (**B1**–**B3**) and in KO/WT/+RIBEYE-C-TG mice (**D1**–**D3**) ((**B1**–**B3**): Manders’ coefficient 1 (green signals overlapping with red signals, M1): 0.7507, Manders’ coefficient 2 (red signals overlapping with green signals, M2): 0.7518 (N = 4, n = 41); (**D1**–**D3**): Manders’ coefficient M1 (green signals overlapping with red signals): 0.7462; Manders’ coefficient M2 (red signals overlapping with green signals): 0.6591; N = 4, n = 47); N, number of mice; n, number of analyzed images. Abbreviations: OPL, outer plexiform layer. Scale bars: 5 μm.

The transgenic RIBEYE-C-TG protein appeared properly localized to the active zones, as judged by double-immunolabeling with mouse monoclonal antibody against the photoreceptor synapse active zone protein RIM2 [35,61,62,63] (Figure 3). In Figure 3, the transgenic RIBEYE-C-TG protein was visualized with rabbit polyclonal antibody against HA (Figure 3(B1,D1)), which is fused to the carboxy-terminus of RIBEYE B-domain (Supplemental Figure S1B). There was no HA signal in retinal synapses of mice that did not contain the RIBEYE-C-TG transgene, i.e., KO/KO mice and KO/WT mice (Figure 3(A1,C1)) emphasizing the specificity of the HA immunosignals in the transgenic RIBEYE-C-TG mice.

**Figure 4:**
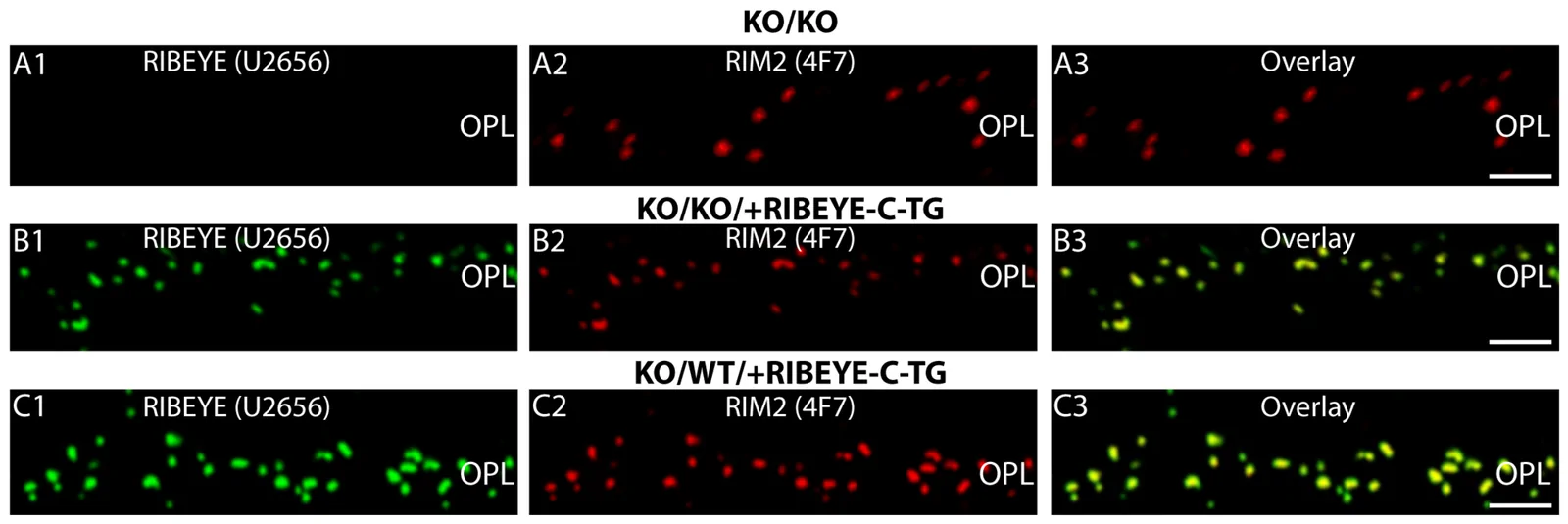
“Transgenic” synaptic ribbons consisting only of RIBEYE-C-TG (formed in RIBEYE KO mice that express RIBEYE-C-TG) are properly localized to the RIM2-containing active zone of photoreceptor synapses in the OPL. Representative images from semi-thin resin retina sections of the indicated mice were immunolabeled with mouse monoclonal antibody against RIM2 (clone 4F7) (**A2**,**B2**,**C2**) and rabbit polyclonal antibody against RIBEYE B-domain (U2656) (**A1**,**B1**,**C1**). Signals from the green channels (**A1**,**B1**,**C1**) and the red channels (**A2**,**B2**,**C2**) were merged in (**A3**,**B3**,**C3**). RIBEYE KO mice do not possess synaptic ribbons, as shown by immunolabeling with polyclonal anti-RIBEYE ((**A1**), see also [12]), although the KO synapses contain active zones, as visualized with mouse monoclonal RIM2 antibody (**A2**). In contrast, RIBEYE-C-TG-expressing RIBEYE KO mice generate synaptic ribbons close to the RIM2-immunolabeled active zone (**B1**). A similar immunolabeling pattern to that in (**B1**–**B3**) can be observed in immunolabeled heterozygous KO/WT mice containing a WT allele, a KO allele, and the RIBEYE-C-TG transgene (**C1**–**C3**), indicating that the WT protein and the transgenic RIBEYE-C-TG protein both assemble into synaptic ribbons. The immunosignals obtained with the rabbit polyclonal RIBEYE B-domain antibody U2656 and with the mouse monoclonal RIM2 antibody clone 4F7 showed a high degree of colocalization in KO/KO/+RIBEYE-C-TG mice and in KO/WT/+RIBEYE-C-TG mice ((**B1**–**B3**): Manders’ coefficient 1 (green signals overlapping with red signals, M1): 0.8135, Manders’ coefficient 2 (red signals overlapping with green signals, M2): 0.7507 (N = 4, n = 45); in (**C1**–**C3**): Manders’ coefficient M1 (green signals overlapping with red signals): 0.7851; Manders’ coefficient M2 (red signals overlapping with green signals): 0.8069; N = 4, n = 45). Abbreviations: OPL, outer plexiform layer; KO, knockout; WT, wild-type; N, number of mice; n, number of analyzed images. Scale bars: 5 μm.

The proper localization of RIBEYE-C-TG-containing synaptic ribbons to the photoreceptor active zone neurotransmitter release site was further confirmed by additional immunolabeling experiments in which transgenic synaptic ribbons were visualized with polyclonal antibodies against RIBEYE B-domain (U2656; [11]) and mouse monoclonal antibodies against RIM2 (clone 4F7; [35]) in the indicated mouse genotypes (Figure 4). Immunolabeled sections were analyzed by confocal microscopy (Figure 4(A1–A3,B1–B3,C1–C3)). These immunolabeling data confirmed the proper localization of the “transgenic” synaptic ribbons consisting of RIBEYE-C-TG at the presynaptic active zone of the photoreceptor ribbon synapses of RIBEYE KO/KO/+RIBEYE-C-TG mice (Figure 4(B1–B3)) and RIBEYE KO/WT/+RIBEYE-C-TG mice (Figure 4(C1–C3)).

Next, we analyzed whether RIBEYE WT protein and RIBEYE-C-TG protein could integrate into the same synaptic ribbon in RIBEYE-C-TG expressing KO/WT RIBEYE mice, i.e., in mice that contain a RIBEYE WT allele and a KO allele (KO/WT/+RIBEYE-C-TG). For this purpose, it was important to discriminate between endogenous mouse RIBEYE protein and the transgenic RIBEYE-C-TG protein. As mentioned above, we used cDNA encoding rat RIBEYE for the generation of the RIBEYE-C-TG mice [26,27]. The rat RIBEYE protein is very similar in amino acid sequence to the mouse RIBEYE protein, but some amino acid sequence differences exist in RIBEYE A-domain, which is relatively divergent between species [2,11]. We exploited these amino acid differences in RIBEYE A-domains of mice and rats in the present study by using the mouse monoclonal RIBEYE A-domain antibody (clone 6F4; [25]). The RIBEYE A-domain antibody 6F4 detects only mouse RIBEYE protein, but not rat RIBEYE protein (Supplementary Figure S2(A,B,C1,C2)). Thus, this monoclonal antibody can discriminate between the untagged endogenous mouse RIBEYE protein and the carboxy-terminally tagged rat RIBEYE transgenic protein (Supplementary Figure S2(A,B,C1,C2)). The endogenous mouse RIBEYE protein can be detected with the 6F4 monoclonal antibody against the RIBEYE A-domain (Supplementary Figure S2(A,B,C1,C2)), and the transgenic rat RIBEYE protein can be specifically detected with antibodies against the protein tags of the transgenic protein (e.g., tRFP-,SNAP-, or HA-tag) (Supplementary Figure S1B).

**Figure 5:**
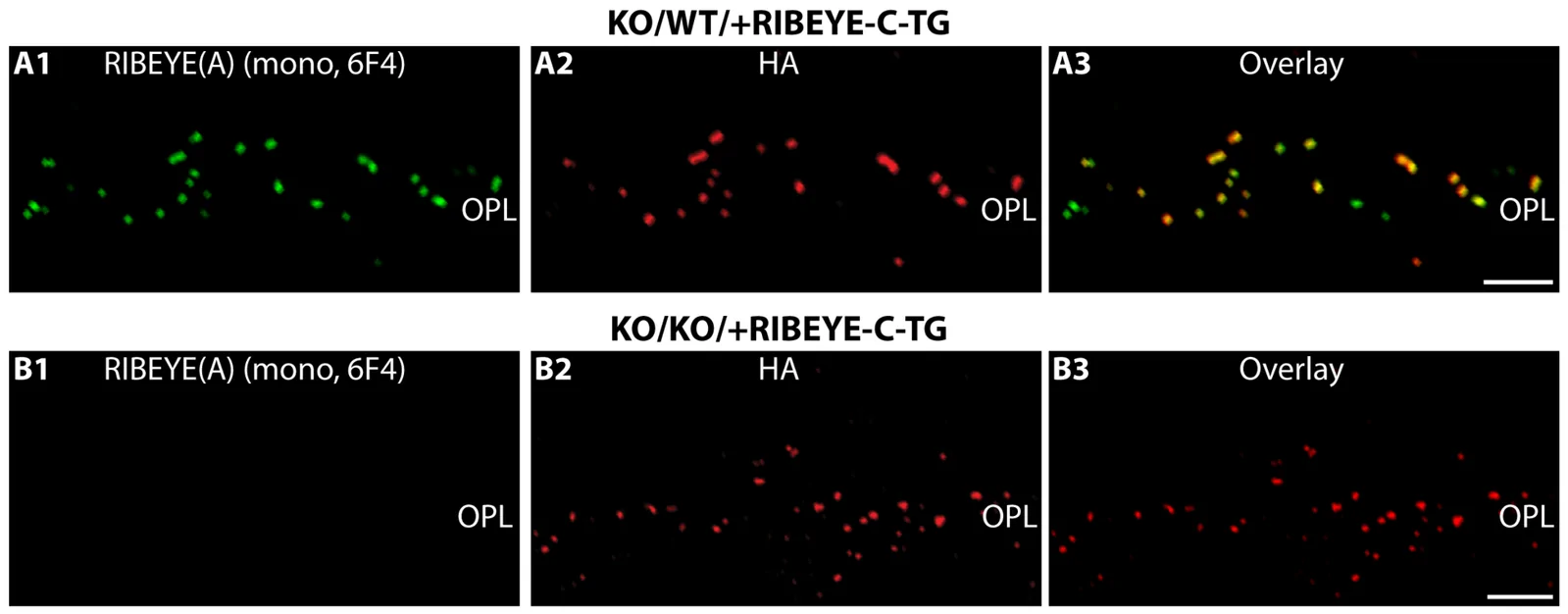
Transgenic RIBEYE-C-TG is incorporated into endogenous synaptic ribbons in heterozygous RIBEYE (KO/WT) mice containing a single WT allele. Representative images of semi-thin retina sections of KO/WT/+RIBEYE-C-TG mice (**A1**–**A3**) and KO/KO/+RIBEYE-C-TG mice (**B1**–**B3**) were double-immunolabeled with rabbit polyclonal antibodies against the HA-tag (**A2**,**B2**) and mouse monoclonal antibody against RIBEYE A-domain (clone 6F4) (**A1**,**B1**). Signals from the green channels (**A1**,**B1**) and the red channels (**A2**,**B2**) were merged in (**A3**,**B3**). In KO/KO/+RIBEYE-C-TG mice, no immunosignal was observed with the RIBEYE A-domain antibody 6F4 (**B1**) because this antibody is specific for mouse RIBEYE (see Supplementary Figure S2(A,B,C1,C2)). The immunosignals obtained with the rabbit polyclonal antibody against the HA-tag and with the mouse monoclonal RIBEYE A-domain antibody clone 6F4 showed a high degree of colocalization in KO/KO/+RIBEYE-C-TG mice in (**A1**–**A3**) (Manders’ coefficient 1 (green signals overlapping with red signals, M1): 0.6647; Manders’ coefficient 2 (red signals overlapping with green signals, M2): 0.9829; N = 4; n = 46). Abbreviations: OPL, outer plexiform layer; KO, knockout; WT, wild type; RIBEYE-C-TG, RIBEYE-C-TG transgene; N, number of mice; n, number of analyzed images. Scale bars: 5 μm.

Based on these findings, we used the mouse monoclonal anti-RIBEYE A-domain antibody (clone 6F4) for the detection of the (untagged) endogenous mouse RIBEYE protein and the rabbit polyclonal antibody against the HA-tag for the visualization of the transgenic, carboxy-terminally tagged RIBEYE protein (Figure 5). As mentioned above, the HA-tag is fused to the carboxy-terminus of the transgenic RIBEYE-C-TG protein (Supplementary Figure S1B) and absent from the endogenous mouse RIBEYE WT protein. Co-labeling of heterozygous KO/WT RIBEYE mice (containing one WT allele and one KO allele) that also express transgenic RIBEYE-C-TG protein demonstrated that RIBEYE WT protein and RIBEYE-C-TG transgenic protein can integrate into the same synaptic ribbon as judged by the co-localization of the two respective immunosignals (Figure 5(A1–A3)). In homozygous RIBEYE KO/KO mice, which also express the RIBEYE-C-TG transgene (KO/KO/+RIBEYE-C-TG) (Figure 5(B1–B3)), only the anti-HA tag antibody produced a synaptic ribbon signal in photoreceptor synapses in the OPL (Figure 5(B2)), whereas the anti-RIBEYE A-domain antibody 6F4 did not generate a synaptic ribbon signal in the OPL (Figure 5(B1)) because the synaptic ribbon in this mouse genotype is exclusively made by the transgenic rat RIBEYE protein, which is not recognized by the 6F4 RIBEYE A-domain antibody (Figure 5(B1)).

Identical findings were observed with antibodies against tRFP-tag and the SNAP-tag of RIBEYE-C-TG (Supplemental Figure S3(A1–A3,B1–B3,C1–C3)). In KO/WT/RIBEYE-C-TG mice, the immunosignal obtained with the tRFP-tag, SNAP-tag, and HA-tag antibodies in the OPL overlapped with the immunosignals obtained with the RIBEYE A-domain 6F4 monoclonal antibody (Supplemental Figure S3(A1–A3,B1–B3,C1–C3)). The RIBEYE A-domain 6F4 monoclonal antibody did not generate a synaptic ribbon signal in the OPL of KO/KO/RIBEYE-C-TG mice because the synaptic ribbons in these mice are exclusively made by rat RIBEYE-C-TG protein (Supplemental Figure S3(D1–D3,E1–E3,F1–F3)). In heterozygous KO/WT mice without a RIBEYE-C-TG transgene, the three different antibodies against the tag proteins (i.e., tRFP, SNAP, HA) did not detect a synaptic ribbon immunosignal in the OPL (Supplemental Figure S3(G1–G3,H1–H3,I1–I3)), further indicating the specificity of the immunolabeling data.

**Figure 6:**
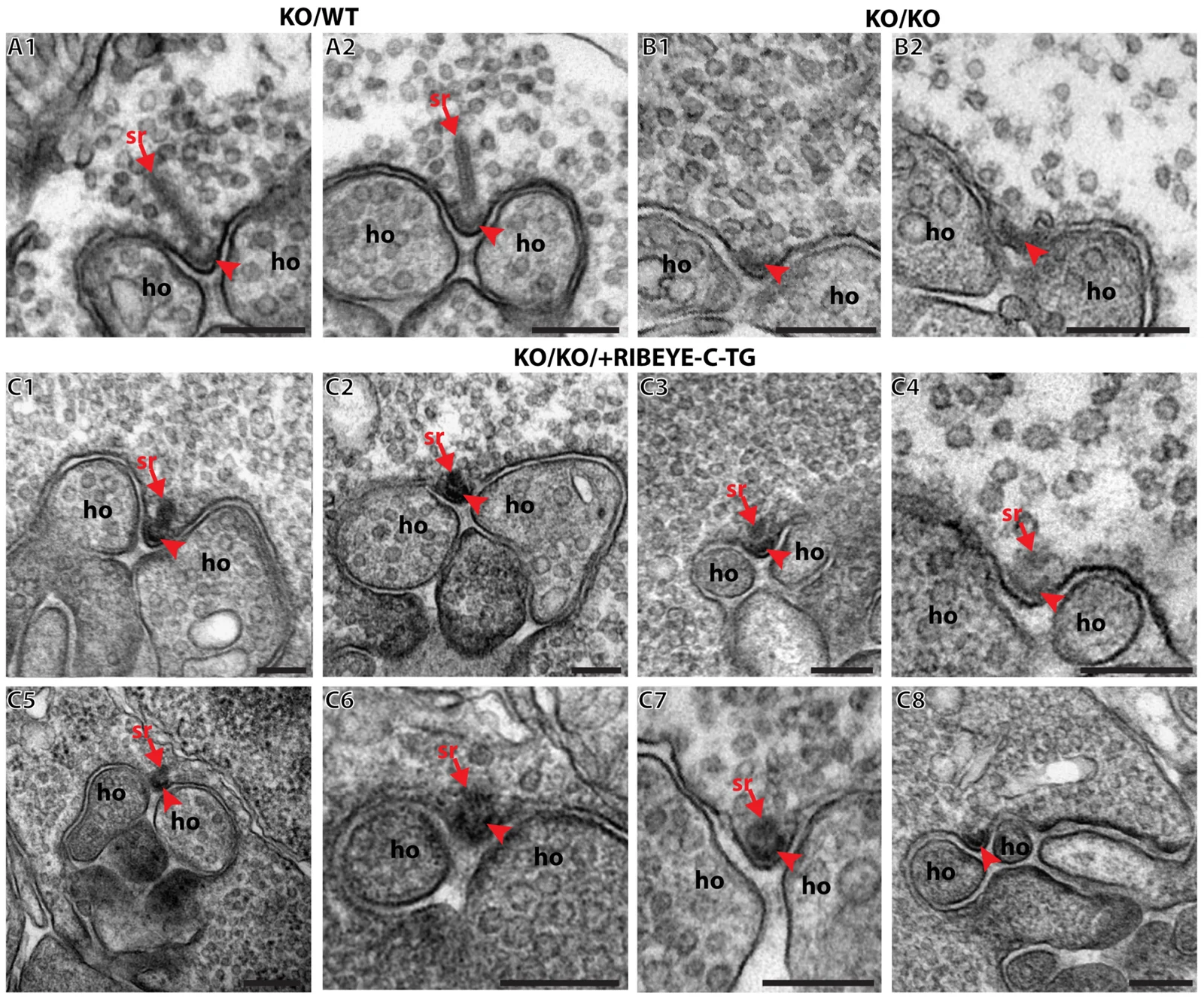
Transgenic RIBEYE-C-TG expression rescues synaptic ribbon formation in photoreceptor synapses of RIBEYE KO mice as analyzed by TEM, but incompletely. Synaptic ribbons are present at the active zone of photoreceptor synapses of heterozygous WT/KO (control) mice (**A1**,**A2**) in their characteristic bar-shaped appearance (**A1**,**A2**). In RIBEYE KO/KO mice, synaptic ribbons were completely absent (**B1**,**B2**), as also previously observed [12]. In contrast, RIBEYE-C-TG-expressing RIBEYE KO mice (KO/KO/+RIBEYE-C-TG) generated synaptic ribbons (**C1**–**C8**). These synaptic ribbons made by RIBEYE-C-TG (**C1**–**C8**) were associated with the presynaptic active zone neurotransmitter release sites but were much shorter in size than in heterozygous KO/WT (control) retinas with a single WT allele (**A1**,**A2**) vs. (**C1**–**C8**); for quantification, see Figure 7. The synaptic ribbons made by RIBEYE-C-TG appeared also partly less electron-dense (**C4**) and sometimes even empty active zones without synaptic ribbons were observed (**C8**). Red arrowheads point to the arciform density (active zone) of rod photoreceptor synapses (**A1**,**A2**,**B1**,**B2**,**C1**–**C8**). In (**C1**–**C7**), red arrows point to short synaptic ribbons in RIBEYE-C-TG-expressing RIBEYE KO/KO/+RIBEYE-C-TG mice. In (**A1**,**A2**), red arrows point to normally sized synaptic ribbons in heterozygous control mice. Abbreviations: sr, synaptic ribbon; ho, postsynaptic dendritic tips of horizontal cells; KO, knockout; WT, wild type; RIBEYE-C-TG, RIBEYE-C-TG transgene. Scale bars: 200 nm.

**Figure 7:**
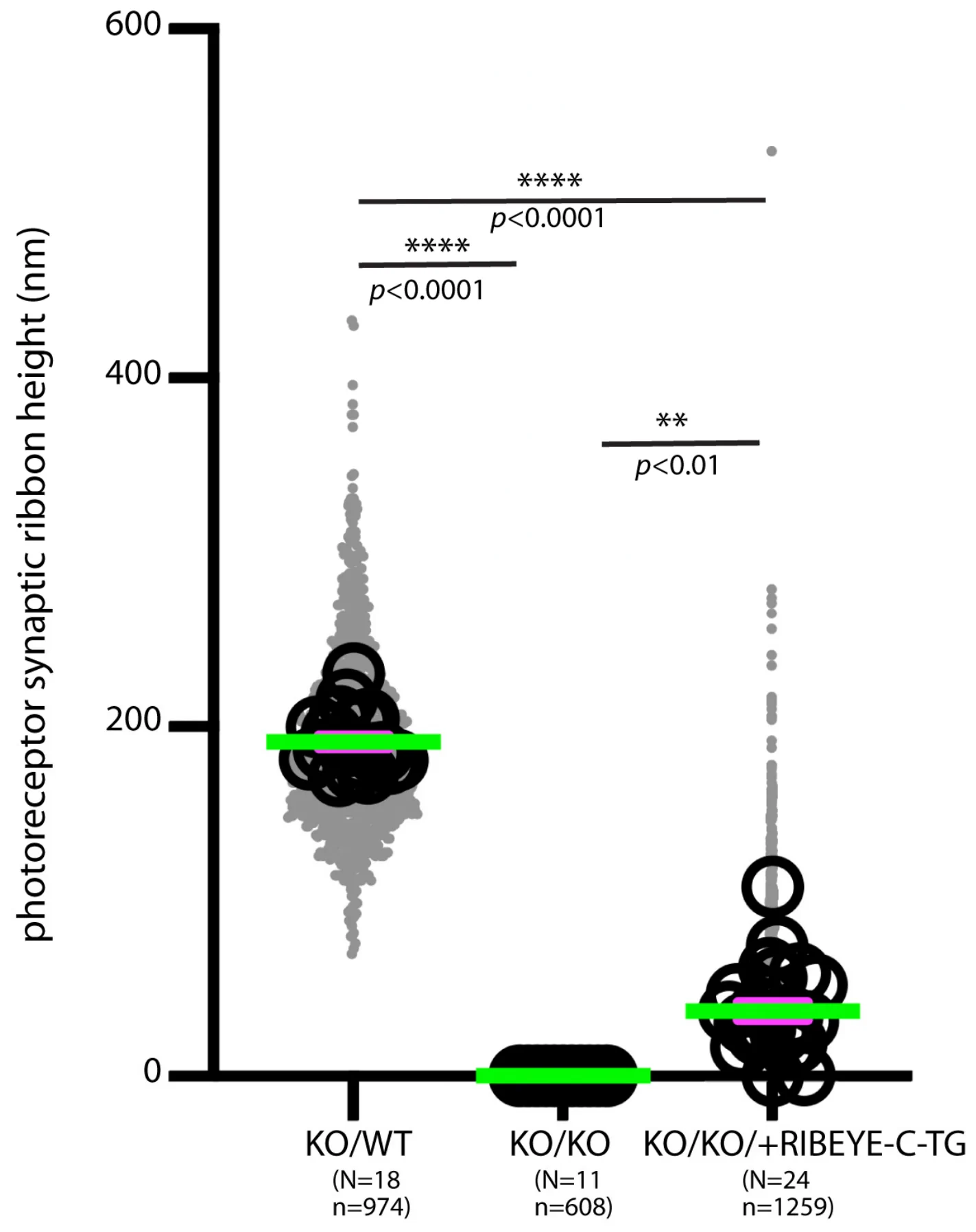
Quantification of photoreceptor synaptic ribbon height in the indicated mouse genotypes. The large non-filled circles indicate the arithmetic mean values of the individual mice, the small grey dots all individual datapoints. Green horizontal bars show the arithmetic means of all individual experiments; error bars are S.E.M. (in magenta). Normal-/non-normal distribution of data was tested with the Kolmogorov–Smirnov test. The mean values of the individual experiments were compared for statistical significance with Kruskal–Wallis ANOVA with Dunn’s post hoc correction for multiple comparisons to analyze the effect of transgenic expression of RIBEYE-C-TG in the KO mice. In RIBEYE KO mice (KO/KO), synaptic ribbons were completely absent from the active zone of photoreceptor synapses, as also previously described [12]. In contrast, synaptic ribbons were observed in RIBEYE-C-TG-expressing KO mice (KO/KO/+RIBEYE-C-TG) (arithmetic mean of photoreceptor synaptic ribbon height in KO/KO/+RIBEYE-C-TG mice: 37.2 nm ± 4.8 nm S.E.M.), but smaller than in the KO/WT reference mice (arithmetic mean of photoreceptor synaptic ribbon height in KO/WT heterozygous reference mice: 191.3 nm ± 3.7 nm S.E.M.). Heterozygous KO/WT mice served as control mice because they contain a single WT allele. *p*-values show adjusted *p*-values (adjusted for multiple comparisons). Abbreviations: N = number of individual mice analyzed; n = number of synapses analyzed. WT, wild-type allele; KO, knockout allele; RIBEYE-C-TG; RIBEYE-C-TG transgene; **, *p* < 0.01; ****, *p* < 0.0001.

Next, we analyzed the synaptic ribbons of photoreceptor synapses in the different mouse genotypes by qualitative and quantitative transmission electron microscopy (Figure 6 and Figure 7). In heterozygous KO/WT mice (which served as control mice), we observed the typical bar-shaped synaptic ribbons, when cross-sectioned (Figure 6(A1,A2)). Synaptic ribbons were completely absent in RIBEYE KO mice (Figure 6(B1,B2)), as already previously demonstrated [12,25]. RIBEYE-C-TG expression in RIBEYE KO mice rescued synaptic ribbon formation in photoreceptor synapses (Figure 6(C1–C8)). But synaptic ribbons were shorter in these mice, and even empty active zones without a synaptic ribbon were observed (Figure 6(C1–C8)). Thus, the synaptic ribbon rescue by RIBEYE-C-TG appeared less efficient than synaptic ribbon formation by untagged RIBEYE WT protein. Heterozygous KO/WT mice served as reference in the experiments (Figure 6(A1,A2)).

The quantification of synaptic ribbon height by TEM in mouse photoreceptor synapses of the indicated genotypes is depicted in Figure 7.

**Figure 8:**
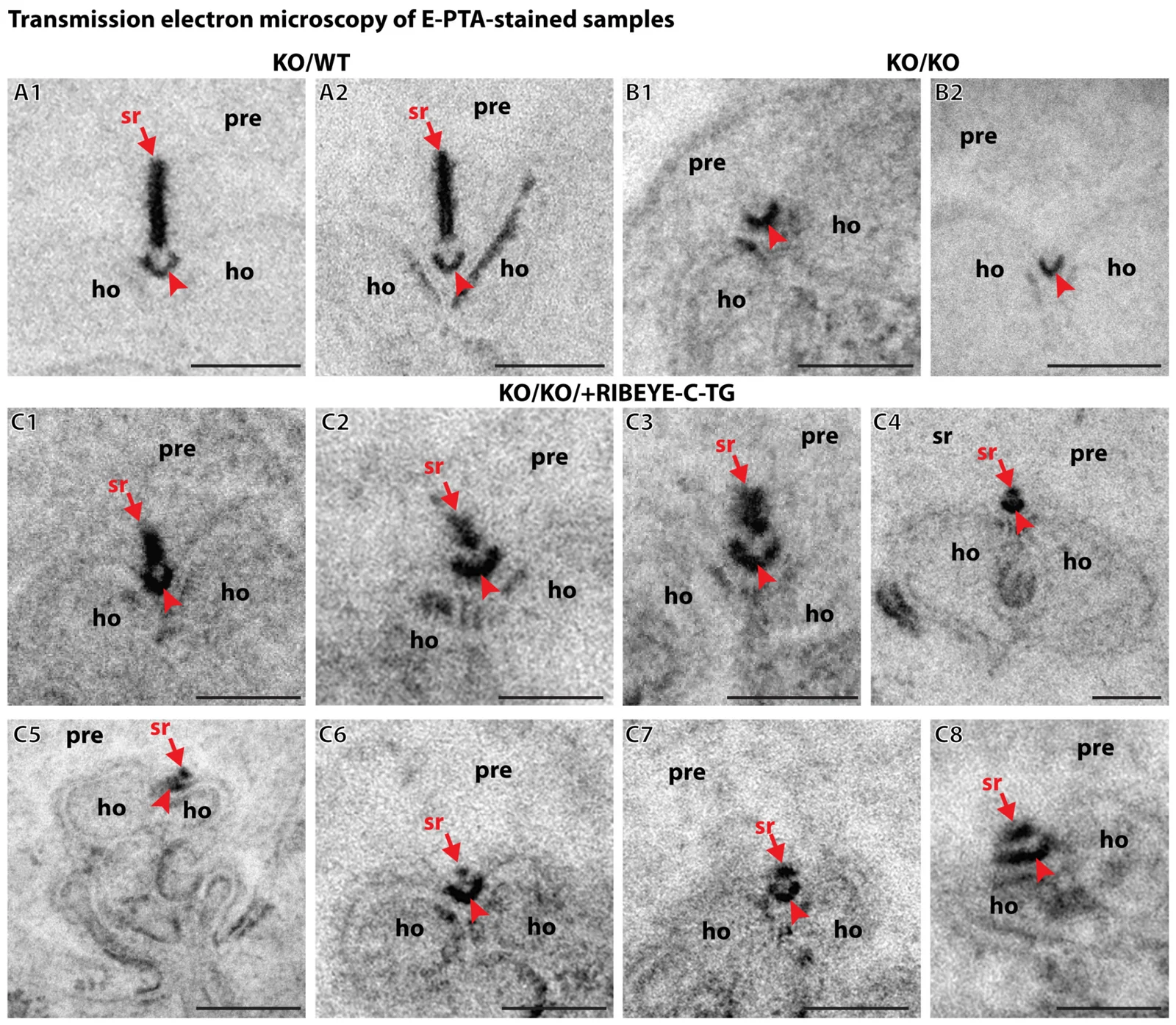
Transgenically expressed RIBEYE-C-TG protein rescues synaptic ribbon formation in rod photoreceptor synapses of RIBEYE KO/KO mice, but incompletely. TEM images were obtained from E-PTA-stained retina samples. Synaptic ribbons in photoreceptor synapses are shown in E-PTA-stained synapses of the indicated genotypes. Please note that only pre- and postsynaptic densities and synaptic ribbons are visualized in TEM by E-PTA staining. Synaptic vesicles and plasma membranes remain largely invisible in E-PTA-stained samples. Synaptic ribbons are clearly present at the active zone of photoreceptor synapses of heterozygous WT/KO (control) mice in their characteristic bar-shaped appearance (**A1**,**A2**). In contrast, synaptic ribbons are completely absent in photoreceptor ribbon synapses of homozygous RIBEYE KO/KO mice (**B1**,**B2**). In RIBEYE-C-TG-expressing RIBEYE KO/KO mice (KO/KO/+RIBEYE-C-TG), synaptic ribbons were formed, but these synaptic ribbons were much smaller in size (**C1**–**C8**) than in the KO/WT control mice (**A1**,**A2**), similar to the data obtained by conventional TEM (Figure 6 and Figure 7). Red arrowheads point to the arciform density (active zone) of photoreceptor synapses (**A1**,**A2**,**B1**,**B2**,**C1**–**C8**). In (**C1**–**C8**), red arrows point to short synaptic ribbons in RIBEYE-C-TG expressing homozygous RIBEYE KO (KO/KO/+RIBEYE-C-TG) mice. In (**A1**,**A2**), red arrows point to normally sized synaptic ribbons in heterozygous control mice. Abbreviations: sr, synaptic ribbon; pre, presynaptic; ho, dendritic tips of horizontal cells; WT, wild type; KO, knockout; RIBEYE-C-G, RIBEYE-C-TG transgene; E-PTA, ethanolic phosphotungstic acid; TEM, transmission electron microscopy. Scale bars: 200 nm.

A similar outcome on the size of rod photoreceptor synaptic ribbons in photoreceptor synapses of the different genotypes was observed by transmission electron microscopy (TEM) of ethanolic phosphotungstic acid (E-PTA)-stained retina samples (Figure 8).

**Figure 9:**
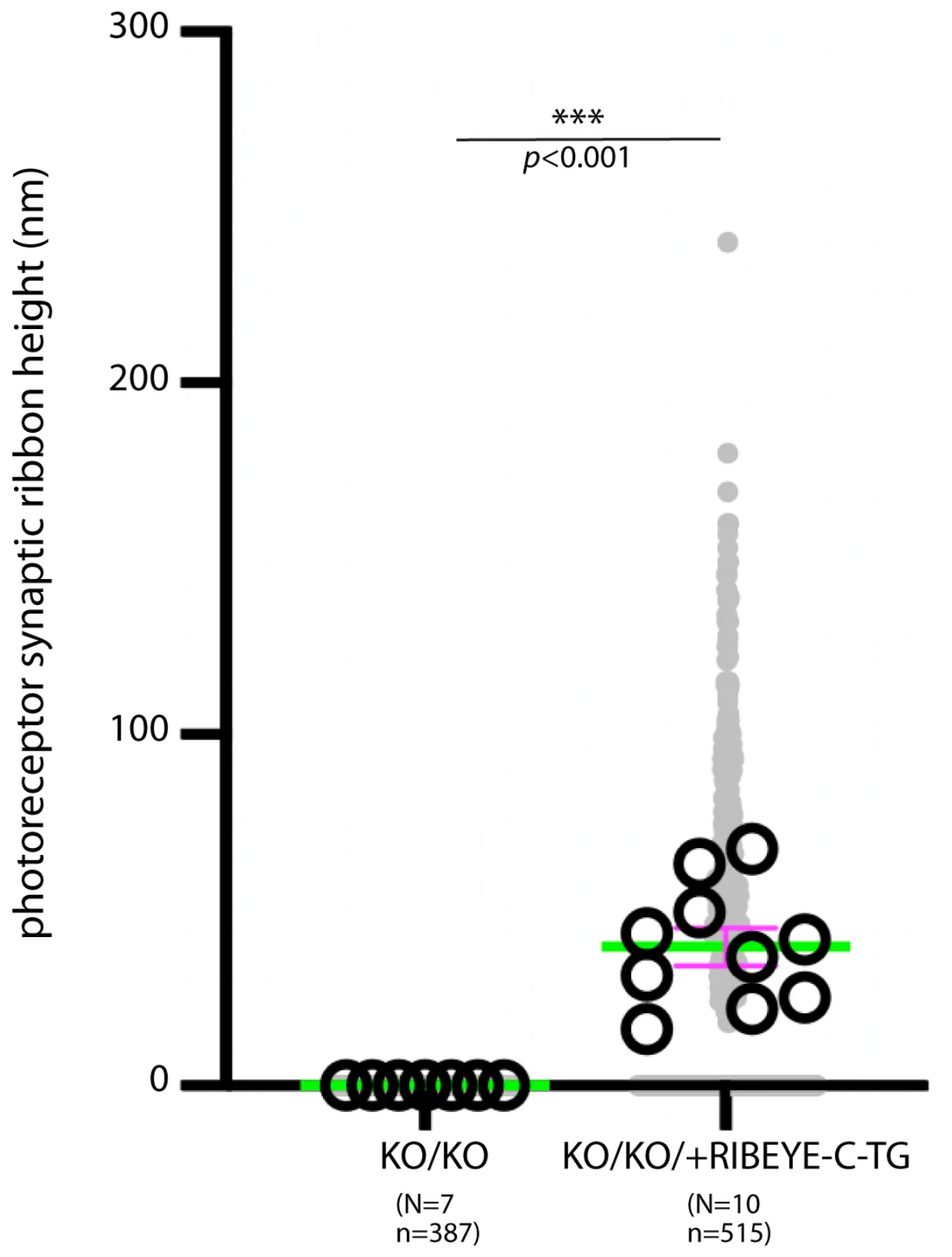
RIBEYE-C-TG expression in RIBEYE KO/KO mice rescues photoreceptor synaptic ribbon formation: quantification of synaptic ribbon height from TEM images of E-PTA-stained retinas. In RIBEYE KO mice (KO/KO), synaptic ribbons were completely absent, as also previously described [12]. In contrast, synaptic ribbons were formed in RIBEYE-C-TG-expressing KO mice (KO/KO/+RIBEYE-C-TG). The height of the synaptic ribbons in KO/KO/+RIBEYE-C-TG mice was quantified as also done in the KO/KO mice. A significant difference in synaptic ribbon height was observed between KO/KO and KO/KO/+RIBEYE-C-TG, similar to that observed by conventional TEM (Figure 6 and Figure 7). The large non-filled circles indicate the arithmetic means of the individual mice, and the small grey dots indicate all individual datapoints/individual synapses. The green horizontal lines show the arithmetic means from all experiments; error bars are S.E.M. (in magenta). Data distribution (i.e., normal vs. non-normal distribution) was tested with the Kolmogorov–Smirnov test. The mean values of the individual experiments were compared for statistical significance with Mann–Whitney U-test. Abbreviations: N, number of individual mice analyzed; n, number of synapses analyzed. WT, wild-type; KO, knockout; ***, *p* < 0.001.

Electron microscopy of E-PTA-stained retina samples provides a high contrast display of synaptic ribbons and pre- and postsynaptic densities, whereas other structures of the photoreceptor synapse remain largely unstained [25,47,50]. Therefore, E-PTA electron microscopy is a particularly suitable additional ultrastructural method for quantitative analyses of synaptic ribbons by eliminating potentially disturbing background signals from other structures within the presynaptic terminal. The quantification of the synaptic ribbon height from the TEM images of E-PTA-stained retinas (Figure 9) showed similar results as observed by conventional TEM (Figure 6 and Figure 7). E-PTA electron microscopy confirmed that expression of RIBEYE-C-TG in RIBEYE KO mice rescued synaptic ribbon formation (Figure 9). E-PTA electron microscopy confirmed that photoreceptor synaptic ribbons were made in KO/KO-RIBEYE-C-TG mice in contrast to KO/KO mice that did not form any synaptic ribbons (Figure 9). These data reaffirm that RIBEYE-C-TG can generate synaptic ribbons in RIBEYE KO mice. The arithmetic mean height of photoreceptor synaptic ribbons in KO/KO+RIBEYE-C-TG mice in E-PTA electron microscopy was 39.6 nm (±5.4 nm S.E.M.). The value obtained for synaptic ribbon height in photoreceptor synapses in KO/KO+RIBEYE-C-TG animals obtained by EPTA electron microscopy was almost identical to the value obtained by conventional transmission electron microscopy (arithmetic mean of ribbon height: 37.2 nm ± 4.8 nm S.E.M.) (Figure 7). These synaptic ribbons in rod photoreceptor synapses of KO/KO/RIBEYE-C-TG mice are considerably shorter than the ones found in the heterozygous KO/WT mice (Figure 7).

**Figure 10:**
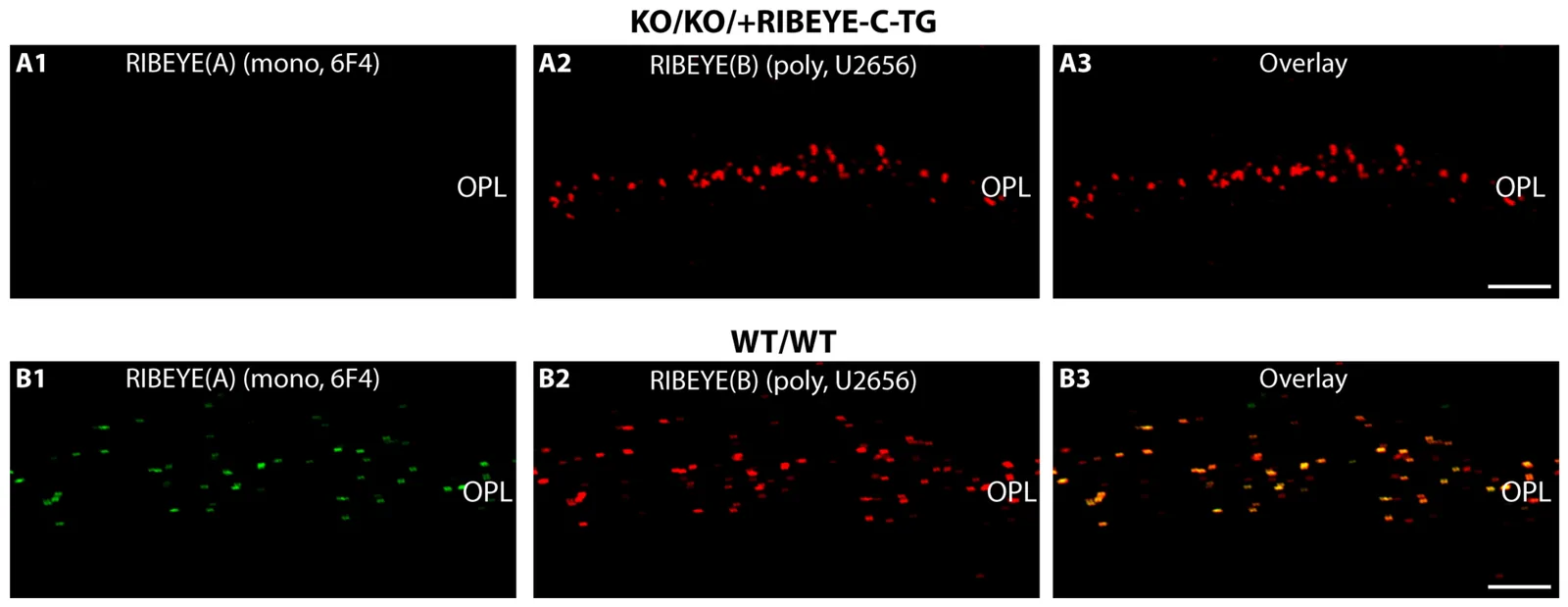
Representative images from semi-thin retina sections from the indicated mouse genotypes double-immunolabeled with rabbit polyclonal antibody against RIBEYE B-domain (U2656) (**A2**,**B2**) and mouse monoclonal RIBEYE A-domain antibody (clone 6F4) (**A1**,**B1**). Signals from the green channels (**A1**,**B1**) and the red channels (**A2**,**B2**) were merged in (**A3**,**B3**). The 6F4 monoclonal antibody detects mouse RIBEYE (**B1**), but not rat RIBEYE encoded by the RIBEYE-C-TG transgene (**A1**). The immunosignals obtained with the mouse monoclonal RIBEYE A-domain antibody clone 6F4 and with the rabbit polyclonal RIBEYE B-domain antibody U2656 showed a high degree of colocalization (Manders’ coefficient 1 [green signals overlapping with red signals, M1]: 0.9733; Manders’ coefficient 2 [red signals overlapping with green signals, M2]: 0.9166; N = 4; n = 41). Abbreviations: OPL, outer plexiform layer; WT, wild type; KO, knockout; RIBEYE-C-TG, RIBEYE-C-TG transgene; N, number of mice; n = number of analyzed images. Scale bar: 5 μm.

We further verified that these synaptic ribbons in the RIBEYE-C-TG-expressing RIBEYE knockout mice were only made by the transgenic RIBEYE-C-TG protein in the absence of the endogenous RIBEYE protein by using the RIBEYE A-domain monoclonal antibody clone 6F4 that detects only mouse RIBEYE but not the transgenic (rat) RIBEYE-C-TG protein. The transgenic synaptic ribbons in the RIBEYE KO were positive for the rabbit polyclonal RIBEYE B-domain antibody (U2656) that labels both mouse and rat RIBEYE (Supplemental Figure S2(A2,B2); Figure 10(A2,B2)) but negative for the mouse monoclonal RIBEYE antibody clone 6F4, which only detects mouse RIBEYE (Figure 10(A2)) but not the transgenic rat RIBEYE-C-TG (Figure 10(A1)).

Thus, the synaptic ribbons in photoreceptor synapses of RIBEYE-C-TG-expressing RIBEYE KO mice were very small in TEM, i.e., much smaller in height than the heterozygous KO/WT reference mice with one WT allele, as consistently shown by both conventional electron microscopy as well as by E-PTA electron microscopy (Figure 6, Figure 7, Figure 8 and Figure 9).

Since synaptic ribbons in photoreceptor synapses were only incompletely rescued by transgenic expression of RIBEYE-C-TG protein in RIBEYE KO mice (Figure 6, Figure 7, Figure 8 and Figure 9), we analyzed RIBEYE-C-TG protein expression in the respective mice by quantitative Western blot (WB) analyses.

**Figure 11:**
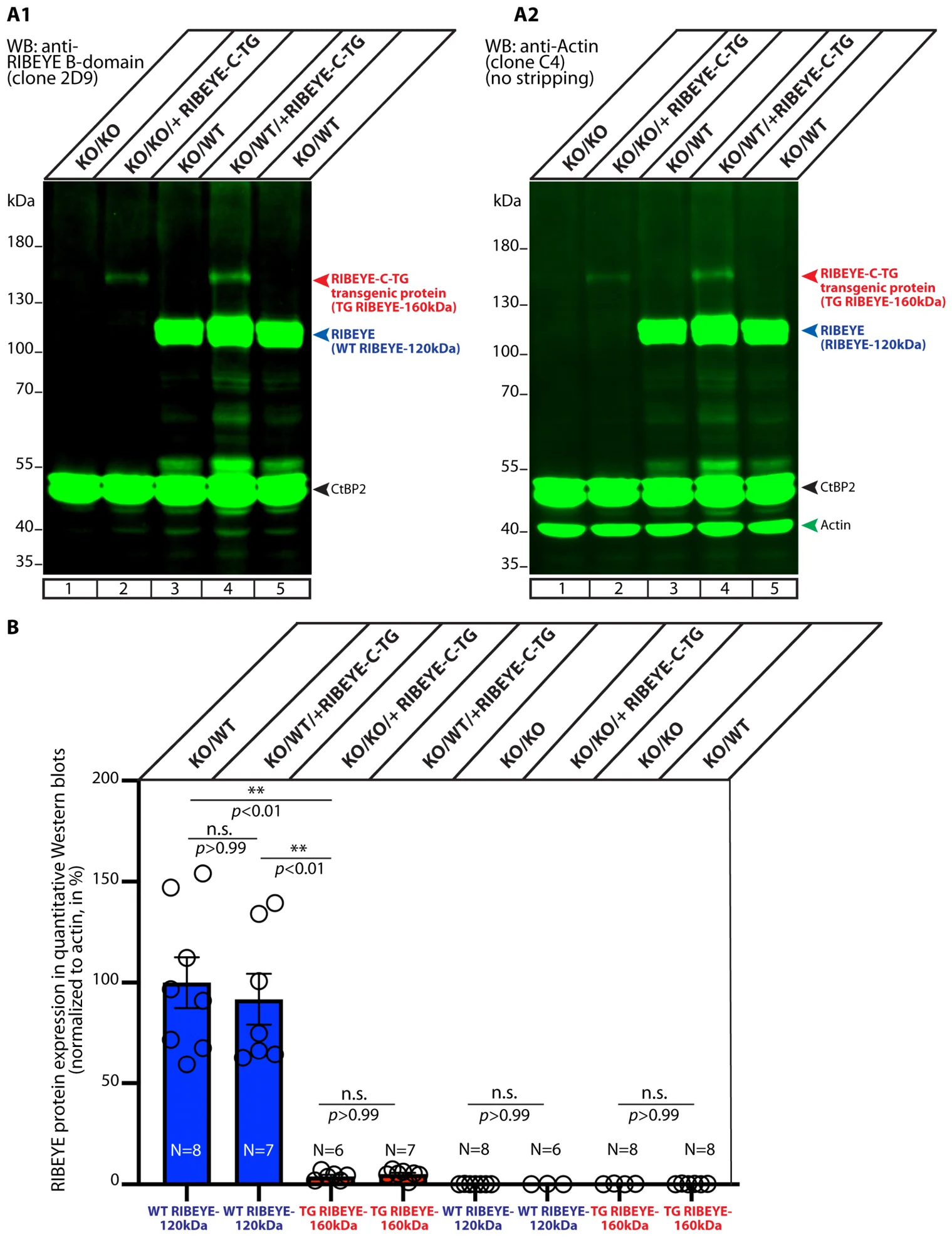
Western blot (WB) analyses to determine the relative expression of transgenic RIBEYE-C-TG protein in the indicated mouse genotypes. (**A1**) Representative WB fluorescence image of retinal lysates obtained from the indicated mice probed with mouse monoclonal anti-RIBEYE B-domain (clone 2D9) primary antibody and chicken anti-mouse Alexa488-conjugated secondary antibody. The protein band marked with a red arrowhead is RIBEYE-C-TG protein at ≈160 kDa (lanes 2, 4); the band marked with the blue arrowhead represents untagged RIBEYE at ≈120 kDa (lanes 3–5); the protein band marked with the black arrowhead represents the ubiquitously expressed CtBP2, which is also detected by the monoclonal antibody against RIBEYE B-domain (clone 2D9) (lanes 1–5). (**A2**) Same blot as shown in (**A1**) but re-incubated (without stripping) with mouse monoclonal antibody against actin (clone C4) and chicken anti-mouse Alexa 488 conjugated secondary antibody. Actin served as loading control and for normalization (green arrowhead in lanes 1–5, (**A2**)). (**B**) Quantification of RIBEYE expression (untagged RIBEYE WT protein at ≈120 kDa and carboxy-terminally tagged RIBEYE-C-TG protein at ≈160 kDa) in the indicated genotypes. The integrated density of the untagged RIBEYE 120 kDa band in KO/WT mice was set to 100%. RIBEYE-C-TG expression at ≈160 kDa in KO/KO/+RIBEYE-C-TG and KO/WT/+RIBEYE-C-TG mice was related to this value. The bar graph shows arithmetic means ± S.E.M. To determine the statistical significance, Brown–Forsythe and Welch ANOVA tests (assuming unequal standard deviations for the different genotypes) and a Dunnett T3 post-hoc test were applied for multiple comparisons, as the data were normally distributed. Abbreviations: WT, wild type; KO, knockout; TG-RIBEYE-160 kDa, RIBEYE-C-TG transgenic protein at ≈160 kDa in WB; WT RIBEYE-120 kDa, wild-type RIBEYE protein at ≈120 kDa in WB; WB, Western blot; S.E.M., standard error of the mean; N = number of mice; **, *p* < 0.01; n.s., non-significant.

Quantitative WB analyses revealed that the RIBEYE-C-TG transgenic allele was considerably lower expressed in comparison to heterozygous WT/KO RIBEYE reference mice, which contain a single copy of the WT allele. As mentioned above, the RIBEYE-C-TG transgenic mice also contain a single copy of the transgene. The expression of the RIBEYE-C-TG protein was only ≈5% of the WT allele as analyzed by quantitative WB analyses (Figure 11).

**Figure 12:**
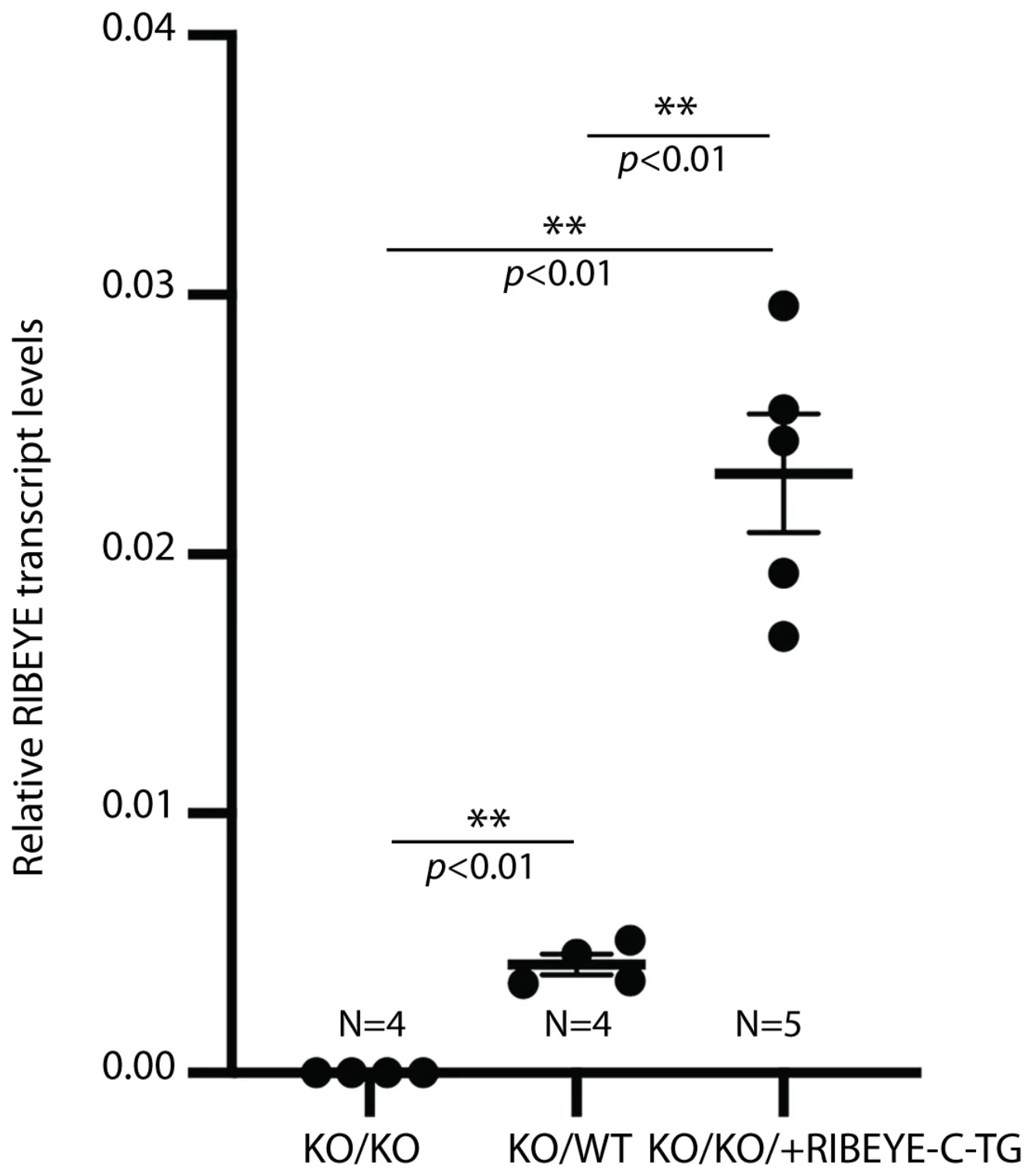
Transgenic RIBEYE-C-TG message is more efficiently transcribed from the transgenic RIBEYE promoter construct in comparison to the endogenous RIBEYE promoter of the RIBEYE gene (quantitative RT-PCR analyses). Determination of RIBEYE transcripts in the indicated mouse genotypes as determined by RT-qPCR. RNA was isolated from the mice of the indicated genotypes and RIBEYE transcripts were detected and analyzed by RT-qPCR for RIBEYE transcripts, as described in “Materials and Methods”. Data were analyzed as RIBEYE transcription standardized to the internal reference gene Actb. The horizontal lines depict the arithmetic means ± S.E.M. Statistical analysis of significance was performed with Brown–Forsythe and Welch ANOVA with Dunnett T3 post hoc correction for multiple comparisons. N, number of mice analyzed for each genotype; **, *p* < 0.01.

Since the protein expression of RIBEYE-C-TG was much lower than RIBEYE WT protein encoded by the endogenous RIBEYE/CtBP2 gene (Figure 11), we searched for the reason for this observation. First, we tested whether the transgenic RIBEYE promoter construct used for the generation of the transgenic mouse might be less active than the RIBEYE promoter in the endogenous RIBEYE/CtBP2 gene. For this purpose, we determined the amount of the RIBEYE transcript in the indicated mouse genotypes by RT-qPCR experiments. RNA was isolated and reverse-transcribed, and the resulting cDNA was analyzed by quantitative PCR using sequence-specific probe-based assays for enhanced specificity (Figure 12). We focused on the comparison between RIBEYE-C-TG expressing KO/KO mice, because the transgene is present in a single copy in KO/KO/+RIBEYE-C-TG mice, and the heterozygous KO/WT mice, which contain a single copy of the WT allele. The RIBEYE KO served as a negative control in these experiments.

RT-qPCR experiments revealed that the transgenic RIBEYE promoter construct was not less active than the endogenous RIBEYE promoter in the RIBEYE/CtBP2 gene in producing RIBEYE transcripts. On the contrary, the transgenic RIBEYE promoter construct generated an even higher RIBEYE transcript level than the endogenous promoter in the RIBEYE/CtBP2 gene (Figure 12; pls. compare KO/KO/+RIBEYE-C-TG with KO/WT). The knockout served as negative control/background control in these experiments. Of note, we also performed control experiments to check for potential contaminations with genomic DNA by using exon-spanning primers demonstrating that genomic DNA was efficiently removed (Supplemental Figure S4).

**Figure 13:**
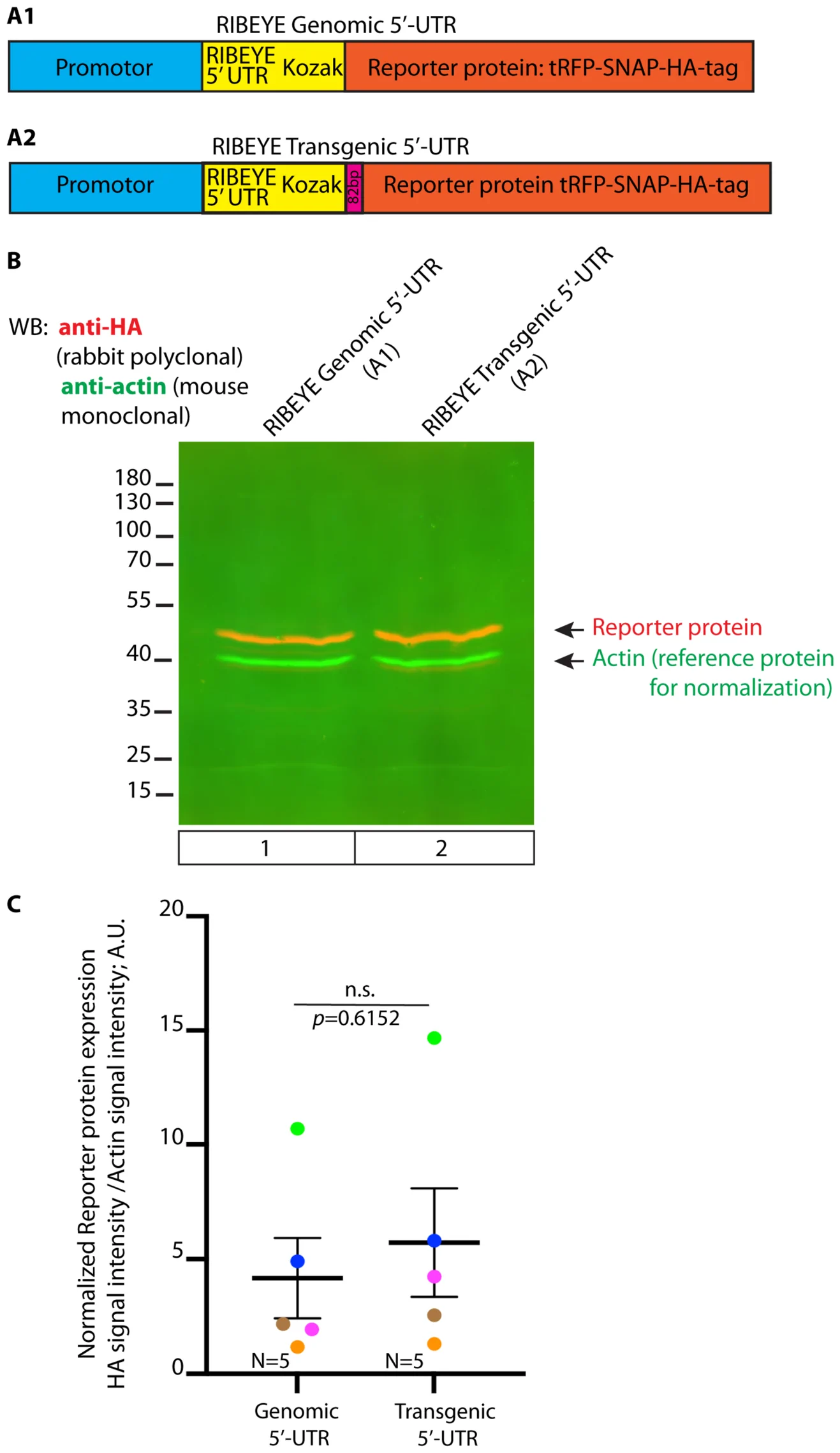
The 5′-UTR of the RIBEYE gene (**A1**) and the 5′-UTR of the RIBEYE transgene (**A2**) do not significantly differ from each other in controlling expression of a reporter protein in a cell transfection assay. The 5′-UTR from the RIBEYE gene (schematically depicted in (**A1**)) and the 5′-UTR of the transgene (schematically depicted in (**A2**)) were placed upstream of a reporter protein-encoding cassette consisting of a triple tag (tRFP, SNAP-tag, and HA-tags) and downstream of a CMV promoter element to analyze the influence of the 5′-UTRs on protein expression. Identical DNA regions are highlighted in identical colors (blue: CMV promoter; yellow: 5′-UTR, orange: reporter protein consisting of tRFP, SNAP-tag, HA-tag). The only difference between the genomic and the transgenic 5′-UTR is an 82 bp insert (purple in (**A2**)) that was generated during cloning of the transgenic vector. Both 5′-UTR regions are similarly effective in driving reporter protein expression (**B**), as also shown by quantitative evaluation (**C**). The additional 82 bp sequence in the 5′-UTR of the transgene does not significantly affect expression of the reporter protein as judged by quantitative WB with anti-HA (to visualize the reporter protein) and anti-Actin (which served as reference protein for quantification and as a loading control) (**B**). (**C**) Quantification of the WB data. The normalized reporter protein expression is depicted. The horizontal lines depict the arithmetic means ± S.E.M. The colored circles show the values of the individual experiments. Statistical significance was analyzed with Welch’s *t*-test; n.s., non-significant.

We also checked whether the 5′-UTR (5′ untranslated region) of the transgenic construct might be less efficient in controlling translation of the respective mRNA than the genomic 5′-UTR using a transfection assay (Figure 13). In this transfection assay, a reporter protein-encoding DNA was placed downstream of the 5′-UTR of the RIBEYE gene or downstream of the 5′-UTR of the RIBEYE-C-TG transgenic construct that contained 82 additional nucleotides for cloning and genotyping purposes (Figure 13(A2)) in comparrison to the RIBEYE gene (Figure 13(A1)). We found that the expression levels of the two mentioned constructs did not differ significantly from each other, indicating that the slightly larger 5′-UTR in the transgenic construct has no negative effect on the translation efficacy (Figure 13B,C). The reporter gene expression was even slightly higher with the transgenic 5′-UTR, although this tendency did not reach statistical significance (Figure 13C).

## 4. Discussion

RIBEYE [11] is the major building block of synaptic ribbons, as demonstrated with RIBEYE KO mice, in which synaptic ribbons are selectively and completely absent [12,64,65], and by quantitative assessment of PXDLS-containing peptide binding to RIBEYE (and CtBP proteins) [66,67]. RIBEYE belongs to the CtBP family of proteins [11,13,68,69]. It consists of a unique amino-terminal A-domain and a carboxy-terminal B-domain that is largely identical to the transcriptional regulator protein CtBP2 [11]. In agreement with the hypothesis of RIBEYE as the major building block of synaptic ribbons, multiple interaction sites were identified in RIBEYE that mediate interaction between different individual RIBEYE proteins [70]. Since the RIBEYE A-domain is the ribbon synapse-specific and unique part of RIBEYE (i.e., absent from CtBP2), and since CtBP2 (including a short variant of CtBP2 that lacks the amino-terminal 20 amino acids) does not form synaptic ribbons [15], the RIBEYE A-domain appeared to be the most critical part for synaptic ribbon formation [11]. More recent studies revealed that the RIBEYE B-domain is also essential to generate synaptic ribbons [25]. Similar to the RIBEYE KO, replacement of RIBEYE B-domain by an irrelevant, unrelated protein (GCaMP3) also resulted in the complete absence of synaptic ribbons in the retina and inner ear [25]. Additionally, replacement of RIBEYE B-domain by the unrelated protein GCaMP3 resulted in the degradation of the full-length RIBEYE protein, emphasizing the importance of the RIBEYE B-domain to stabilize RIBEYE [25]. In the present study, we further pursued the role of the RIBEYE B-domain in synaptic ribbon assembly and analyzed whether carboxy-terminal tagging of the RIBEYE B-domain influences its capability to generate synaptic ribbons.

To address this question, we used a transgenic mouse (RIBEYE-C-TG mouse) that expresses RIBEYE with a large protein triple-tag (tRFP, SNAP-tag, and HA-tag), fused in-frame to the carboxy-terminus of the RIBEYE B-domain (RIBEYE-C-TG protein). The triple tag consisting of tRFP, SNAP-tag, and HA-tag was initially designed for the transgenic RIBEYE-C-TG mice to enable multiple applications. The tRFP-tag and the SNAP-tag would enable imaging in the living retina under native, physiological conditions in the living tissue. Additionally, the SNAP tag would allow versatile labeling with different substrates for multiple, continuously growing applications (e.g., [71,72,73,74,75]). The HA-tag was destined to make the transgenic protein readily detectable by standard immunohistochemical procedures, as shown in the present study. The transgenic RIBEYE-C-TG mouse was cross-bred with RIBEYE KO mice to finally obtain RIBEYE KO mice that only express transgenic, carboxy-terminal-tagged RIBEYE-C-TG, but not endogenous, untagged RIBEYE. We used this KO/KO/+RIBEYE-C-TG mouse model to address the impact of carboxy-terminal tagging of the RIBEYE B-domain on synaptic ribbon formation.

With confocal light microscopy of immunolabeled retina sections, we found that RIBEYE-C-TG indeed rescued synaptic ribbon formation in RIBEYE KO mice. Also, electron microscopy confirmed that synaptic ribbons were made by RIBEYE-C-TG in the KO mice, which were anchored to the active zone and associated with synaptic vesicles. But synaptic ribbons made by RIBEYE-C-TG protein were much smaller in size than synaptic ribbons formed by the untagged RIBEYE WT protein. Both transmission electron microscopy of standard EM samples and of E-PTA-stained EM samples consistently showed a strongly decreased size (height) of photoreceptor synaptic ribbons in RIBEYE-C-TG expressing KO mice. What could be the reason for the incomplete rescue of synaptic ribbon deficiency in the RIBEYE knockout by RIBEYE-C-TG protein?

At the protein level, the transgenic carboxy-terminally tagged RIBEYE-C-TG protein displayed a much lower level of expression than the untagged RIBEYE protein in control retinas (≈5% of the untagged protein), as assessed by comparative quantitative Western blot analyses of retinal lysates from RIBEYE-C-TG-expressing RIBEYE knockout mice and heterozygous KO mice with one WT allele and a KO allele (Figure 11). The transgenic RIBEYE-C-TG mouse contains a single copy of the transgene (Supplemental Figure S1, panel A). Therefore, the comparison with heterozygous KO/WT RIBEYE mice, which contain a single copy of the RIBEYE WT allele, is the most appropriate reference for the KO/KO/+RIBEYE-C-TG mice.

In previous mouse and zebrafish studies, the expression levels of RIBEYE were found to correlate with the size of synaptic ribbons in a mass-equilibrium-law-like manner: the more RIBEYE is expressed, the larger synaptic ribbons were made and vice versa: the less RIBEYE is available, the smaller the synaptic ribbons [25,65,76]. Therefore, the decreased size of synaptic ribbons in RIBEYE-C-TG-expressing RIBEYE KO/KO mice could be explained by the low expression of RIBEYE-C-TG. But why is the expression level of the transgenic RIBEYE-C-TG protein, tagged at the carboxy-terminus of RIBEYE B-domain, so low in comparison to untagged RIBEYE protein?

Several reasons could principally play a role. First, the ≈5 kb promoter element used for the generation of the transgene might be less active than the endogenous promoter of the RIBEYE/CtBP2 gene. We excluded this possibility by showing that the promoter of the transgenic construct is even more active than the endogenous RIBEYE promoter of the RIBEYE/CtBP2 gene, as demonstrated by RT-qPCR experiments (Figure 12). The reason for the stronger activity of the transgenic RIBEYE promoter construct in comparison to the endogenous RIBEYE promoter in the RIBEYE/CtBP2 gene remains to be elucidated. In the RIBEYE/CtBP2 gene, the RIBEYE promoter is placed in the intron downstream of the CtBP2-specific first coding exon [11,14]. Possibly, the RIBEYE promoter in the RIBEYE/CtBP2 gene on mouse chromosome 7 is suppressed by additional upstream genomic elements. CtBP2 is ubiquitously expressed in most eukaryotic cells of vertebrates, whereas RIBEYE expression is limited to synaptic ribbon-forming cells. Thus, in the RIBEYE/CtBP2 gene, transcription of RIBEYE might be negatively influenced by upstream genomic sequences to suppress ectopic expression of RIBEYE, all of which are absent in the RIBEYE-C-TG transgenic construct. However, irrespective of these speculations on the underlying mechanisms, the RT-qPCR experiments clearly showed that the transgenic RIBEYE promoter is not less efficient than the genomic RIBEYE promoter and that a decreased promoter activity is clearly not the reason for decreased RIBEYE-C-TG protein levels.

A second possible explanation could be that the 5′-UTR of the transgenic construct, which is nearly identical to the 5′-UTR of the RIBEYE/CtBP2 gene (except for a stretch of 82 bp inserted during cloning of the transgene), might negatively affect translation efficiency. This second theoretical possibility could be also excluded: In a reporter expression assay, in which we compared the impact of the two slightly different 5′-UTRs, we did not observe any difference in expression of a reporter protein. The protein levels of the reporter protein were even slightly higher with the longer 5′-UTR of the transgene, although this tendency did not reach statistical significance. In summary, we did not find any indication that differences in the promoter or 5′-UTR could explain the decreased expression levels of RIBEYE-C-TG. Third, as mentioned, the RIBEYE-C-TG transgene is encoded by rat RIBEYE and the endogenous protein by mouse RIBEYE. But this species difference is unlikely to play a major role in the incomplete rescue of synaptic ribbon deficiency for several reasons: (i) mouse and rat RIBEYE are highly similar and share 97.3% identical amino acids (rat RIBEYE: AF222712; mouse RIBEYE: NM_001170744), and (ii) rat and mouse photoreceptor synaptic ribbons have very similar sizes [25,40,77].

We favor a fourth possibility for the decreased protein levels of RIBEYE-C-TG. This proposal is based on recent structural and biochemical data [17,18,19,20,21,78,79], as discussed below. Our quantitative Western blot experiments demonstrated that the carboxy-terminally tagged RIBEYE-C-TG protein is less stable than the untagged RIBEYE WT protein (Figure 11). Obviously, the bulky protein tag that was fused to the carboxy-terminus of RIBEYE B-domain imposes a stability problem on RIBEYE with consequences for the assembly of synaptic ribbons, possibly at multiple levels of the RIBEYE-based synaptic ribbon complex. Recently, the tetrameric structure of CtBP2 was solved [17,18,19,20,21,79]. The CtBP dehydrogenase core domain, which was used for the high-resolution structure of CtBP2, is identical to the corresponding region of RIBEYE B-domain [18,19,20,21]. Tetramer formation of RIBEYE B-domain within a synaptic ribbon-like complex in transfected cells has also been directly demonstrated by cryo-EM in a very recent study [78].

In the crystal-/cryo-structure of the CtBP2 tetramer, the carboxy-termini of all four individual CtBP2 subunits are located very closely to each other at the dimer-dimer interaction interface of the individual subunits in the center of the tetramer (6wkw.pdb; [19]). At this location within the internal interaction interface, a bulky tag at the carboxy-terminus of CtBP2/RIBEYE B-domain will likely impair tetramer formation by disrupting the interface of the CtBP2 tetramer. Considering the size of the triple tag, consisting of tRFP-, SNAP-, and HA-tags, which is nearly as big as RIBEYE B-domain itself, this appears to be a reasonable assumption.

Recently, a pioneering structural work of Liu and colleagues [78] provided an additional possibility on how carboxy-terminal tagging of RIBEYE B-domain could interfere with the assembly of synaptic ribbons. In the study by Liu et al., RIBEYE B-domain tetramers were shown to assemble into higher-order tight filaments (B′ filaments, [78]). These helical filaments formed by RIBEYE B-domain (B′ filaments) were suggested to function as the basic units of synaptic ribbons [78] and could assemble into sheet-like structures [78]. Carboxy-terminal tagging of RIBEYE B-domain could interfere with the assembly of these B′ filaments and B′ sheets. The study by Liu et al. [78] demonstrated that the carboxy-termini of the RIBEYE B-domains in these filaments are in close proximity between neighboring RIBEYE B-domain tetramers (9WBL.pdb, [78]), raising the possibility that a large carboxy-terminal tag fused to RIBEYE B-domain could disrupt these B′ filament assemblies.

Thus, we propose that the bulky tag fused with the carboxy-terminus of RIBEYE B-domain interferes either with the tetramerization of RIBEYE B-domain and/or with the formation of RIBEYE-B-domain B′ filaments, both of which likely represent central building blocks of synaptic ribbons.

The bulky triple tag of RIBEYE-C-TG might not only inhibit/prevent RIBEYE B-domain tetramer formation and the assembly of tetramer-containing B′ filaments but could also lead to a destabilization of the RIBEYE molecule itself, as observed in the present study by quantitative Western blotting analyses (Figure 11). In support of this hypothesis, Mani-Telang et al. [79] showed that lack of NADH binding (which is also essential for tetramerization; see [18,19,20,21]) led to a decreased expression of *Drosophila* CtBP [79]. Currently, we cannot discriminate between these different possibilities. Future investigations have to clarify which mechanisms contribute most to the impaired capability of carboxy-terminally tagged RIBEYE B-domain in the making of synaptic ribbons, i.e., whether structural/steric problems of ribbon assembly resulting from the carboxy-terminal tagging or decreased RIBEYE protein levels are more important, or a combination of both mechanisms. Several mechanisms could contribute at the same time, and the different mechanisms could depend on each other, as discussed above. For the latter aspect of protein stability, additional mechanisms, e.g., a potential co-translational misfolding of carboxy-terminally tagged RIBEYE/altered quality-control triage and/or an impact of the carboxy-terminal tag on translation efficiency including RNA secondary structure, different codon usage, and tRNA abundance could also play a role. These possibilities have to be evaluated by future investigations.

## Figures and Tables

**Table 1:** Primary antibodies.

Antibody	Source	References	Dilution
RIBEYE(B)-domain (rabbit polyclonal, U2656)	Lab-made (Knockout-verified)	[11,12]	1:1000 (IF) 1:1000 (WB)
RIBEYE(B)-domain (mouse monoclonal, clone 2D9)	Lab-made (Knockout-verified)	[25,28]	1:1000 (IF) 1:500 (WB)
RIBEYE(A)-domain (mouse monoclonal, clone 6F4)	Lab-made (Knockout-verified)	[25]	1:1000 (IF) 1:500 (WB)
Synaptic vesicle glycoprotein 2 (SV2) (mouse monoclonal antibody IgG1; cell culture supernatant), RRID: AB_2315387	Developmental Studies Hybridoma Bank (DSHB), Univ. Iowa, Iowa City, IA, USA.	[29]	1:50 (IF)
Hemagglutinin (HA) (rabbit polyclonal antibody), ab9110 RRID: AB_307019	Abcam (via VWR International GmbH, Darmstadt, Germany)	[30,31]	1:500–750 (IF) 1:1000 (WB)
Mammalian brain spectrin 240/235 (rabbit polyclonal antibody), ab992.	Chemicon International, Temecula, CA, USA	[32,33,34]	1:1000 (WB)
RIM2 (mouse monoclonal antibody, clone 4F7)	Lab-made (Knockout-verified)	[35]	1:200–400 (IF)
tRFP (rabbit polyclonal antibody, purified IgG), AB233 RRID: AB_2571743	Evrogen (via BioCat GmbH, Heidelberg, Germany)	[36,37]	1:1000 (IF)
SNAP-tag (antigen affinity-purified rabbit polyclonal antibody), P9310S RRID: AB_10631145	New England Biolabs (NEB) Frankfurt am Main, Germany	[38]	1:1000 (IF)
Actin (mouse monoclonal antibody, clone C4), #1501R RRID: AB_2223041	Millipore #1501R (Merck KGaA, Darmstadt, Germany)	[39]	1:1000 (IF) 1:1000 (WB)

IF, immunofluorescence microscopy; WB, Western blot.

**Table 2:** Secondary antibodies.

Antibody	Source	Dilution
Chicken anti-mouse-Alexa488	Invitrogen Molecular Probes, A-21200 (via Thermo Fisher Scientific, Life Technologies GmbH, Darmstadt, Germany)	1:1000 (IF) 1:5000 (WB)
Donkey anti-rabbit-Alexa568	Invitrogen, Molecular Probes, A-10042 (via Thermo Fisher Scientific, Life Technologies GmbH, Darmstadt, Germany)	1:1000 (IF) 1:5000 (WB)
Chicken anti-rabbit-Alexa488	Invitrogen, Molecular Probes, A-21441 (via Thermo Fisher Scientific, Life Technologies GmbH, Darmstadt, Germany)	1:1000 (IF) 1:5000 (WB)
Donkey anti-mouse-Alexa568	Invitrogen, Molecular Probes, A-10037 (via Thermo Fisher Scientific, Life Technologies GmbH, Darmstadt, Germany)	1:1000 (IF)
Donkey anti-mouse-Alexa488	Invitrogen, Molecular Probes, A-21202 (via Thermo Fisher Scientific, Life Technologies GmbH, Darmstadt, Germany)	1:1000 (IF)
Donkey anti-rabbit-Alexa488	Invitrogen, Molecular Probes, A-21206 (via Thermo Fisher Scientific, Life Technologies GmbH, Darmstadt, Germany)	1:1000 (IF)
Goat anti-mouse-peroxidase (POX)	Sigma, Taufkirchen, Germany, A3676	1:5000 (WB)
Goat anti-rabbit-peroxidase (POX)	Sigma, Taufkirchen, Germany, A6154	1:5000 (WB)

IF, immunofluorescence microscopy; WB, Western blot.

## Data Availability

The original contributions presented in this study are included in the article. Further inquiries can be directed to the authors.

## Supplementary Materials

The following supporting information can be downloaded at: https://www.mdpi.com/article/10.3390/cells15171583/s1. Supplemental Figure S1. (A) A single copy of the transgene is present in the RIBEYE-C-TG transgenic mouse line. The mouse offspring generated from breedings between a transgenically positive parent animal and a non-transgenic parent animal for the presence of the RIBEYE-C-TG transgene in the respective offspring. 47 litters from 19 breeder pairs were analyzed. Nearly exactly 50% of offspring from these litters were transgene-positive demonstrating that a single transgenic copy is present in the transgenic animals. (B) Schematic depiction of the transgene encoding RIBEYE with the indicated protein tag fused to the carboxy-terminus of RIBEYE B-domain. (C) In contrast to native cryostat sections, the tRFP protein tag of RIBEYE-C-TG is not fluorescent in semi-thin epoxy resin sections. Native cryostat sections (A,B) and semi-thin epoxy resin sections (C, after resin removal) from the indicated RIBEYE-C-TG-positive mice were analyzed for tRFP fluorescence in the red channel (excitation with the 561 nm laser line; A1,B1,C1) and for RIBEYE immunosignals, obtained with rabbit polyclonal antibody against RIBEYE B-domain (U2656) and donkey anti-rabbit Alexa488 secondary antibody in the green channel (excitation with the 488 nm laser line; A2,B2,C2). Signals from the individual channels were overlaid in (A3,B3,C3). The endogenous fluorescence of the tRFP protein tag of the RIBEYE-C-TG is lost in the resin-embedded epoxy resin sections (C1). Abbreviations: Tg negative, transgene negative; Tg positive, transgene positive; RIBEYE-C-TG, RIBEYE-carboxy-terminally tagged transgenic protein; negative ctrl, negative control. Supplemental Figure S2. The monoclonal antibody against RIBEYE-A-domain (6F4) detects RIBEYE in a species-specific manner. (A,B) The monoclonal RIBEYE A-domain antibody 6F4 detects RIBEYE in the mouse retina (A1), but not in the rat retina (B1), as shown by immunolabeling of rat and mouse retina cryostat sections (A,B). The rabbit polyclonal antibody against RIBEYE B-domain (U2656) detects synaptic ribbons in the OPL of the mouse retina (A2) and in the OPL of the rat retina (B2) demonstrating that synaptic ribbons were present in both species (positive control). (A3) and (B3) are phase contrast images of the respective sections. In (A4) and (B4) all respective single channels were merged. (C) WB experiments also confirmed that the 6F4 antibody does not detect rat RIBEYE in lysates of the rat retina (lane 3, C1). Rat RIBEYE is clearly present in the rat retinal lysate as demonstrated with the mouse monoclonal antibody against RIBEYE B-domain (clone 2D9, lane 2, C1) and rabbit polyclonal antibody against RIBEYE B-domain (U2656; lane 1, C1). In C2, the same lanes as shown in C1 were incubated with rabbit polyclonal antibody against brain spectrin. Brain spectrin served as loading control. The WB was analyzed with a BioRad ChemiDoc XRS system and with ECL detection. The asterisks (*) marks a degradation product of RIBEYE/CtBP2 that is detected by the U2656 antibody. Abbreviations: AA, acrylamide; OPL, outer plexiform layer; WB, Western blot. Scale bars: 5 μm. Supplemental Figure S3. Transgenic RIBEYE-C-TG is incorporated into endogenous synaptic ribbons in heterozygous RIBEYE (KO/WT) mice containing a single WT allele. Representative image of semi-thin retina sections of KO/WT/+RIBEYE-C-TG mice (A,B,C), KO/KO/+RIBEYE-C-TG mice (D,E,F) and KO/WT mice (G,H,I), double-immunolabeled with rabbit polyclonal antibodies against the HA-tag (A2,D2,G2)/SNAP-tag (B2,E2,H2)/tRFP (C2,F2,I2) and mouse monoclonal antibody against RIBEYE A-domain (clone 6F4) (A1,B1,C1,D1,E1,F1,G1,H1,I1). Signals from the red and green channels were overlaid in the right figure panels (A3,B3,C3,D3,E3,F3,G3,H3,I3). In KO/KO/+RIBEYE-C-TG mice, no immunosignal was observed with the RIBEYE A-domain antibody 6F4 (D1,E1,F1) because the 6F4 monoclonal antibody is specific for mouse RIBEYE and does not detect transgenic rat RIBEYE (see Suppl. Figure S2). In KO/WT mice, no immunosignal was observed with the antibodies against the HA-tag (G2), SNAP-tag (H2) and tRFP-tag (I2) because no carboxy-terminal tagged RIBEYE-C-TG is present in these mice demonstrating the specificity of the immunolabeling data obtained with the tag antibodies. Immunosignals generated by the rabbit polyclonal antibody against the HA-tag (A2)/SNAP-tag (B2)/tRFP (C2) and by the mouse specific monoclonal RIBEYE antibody clone 6F4 (A1,B1,C1) show a high degree of colocalization in KO/WT/+RIBEYE-C-TG mice. (A1–A3: Manders’ coefficient 1 (green signals overlapping with red signals, M1): 0.8080; Manders’ coefficient 2 (red signals overlapping with green signals, M2): 0.8508 (N = 4; n = 52); B1–B3: Manders’ coefficient 1 (green signals overlapping with red signals, M1): 0.9251; Manders’ coefficient 2 (red signals overlapping with green signals, M2): 0.7841 (N = 4; n = 46); C1–C3: Manders’ coefficient 1 (green signals overlapping with red signals, M1): 0.8232; Manders’ coefficient 2 (red signals overlapping with green signals, M2): 0.7736 (N = 4; n = 50). Abbreviations: OPL, outer plexiform layer; KO, knockout; WT, wild-type; RIBEYE-C-TG, RIBEYE-C-TG transgene; N, number of mice; n, number of analyzed images. Scale bars: 5 μm. Supplemental Figure S4. Genomic DNA is efficiently removed by DNase digestion. Documentation of removal of genomic DNA of the indicated samples by using primers located in different exons of the actin gene separated by an intron. For this purpose, we used the β-actin (Actb)-specific probe-based HEX dye-conjugated assay (MmPT39a.22214843g, Integrated DNA Technologies, Coralville, IO, USA) to discriminate between cDNA and genomic DNA. If a genomic DNA contamination is present, an amplicon of 272 bp is observed which includes the 125 nucleotide long spanning intron; if only cDNA is present an amplicon of 125 bp is generated. After treatment with DNase (D; in lanes 2, 4, 6, 8, 11, 13, 15, 17, 20, 22, 24, 26, 28), the 272 bp PCR band indicating contaminating genomic DNA was completely absent in all samples indicating efficient removal of genomic DNA by the applied DNase-treatment. Lanes 1, 3, 5, 7, 10, 12, 14, 16, 19, 21, 23, 25, 27 show the same samples but before treatment with DNase. In lanes 9 and 18 DNA size standards are shown. Abbreviations: D; DNase-treated sample; gDNA, genomic DNA; cDNA, copy DNA; HEX, hexachlorfluoresceine; KO, knockout; WT, wild-type; RIBEYE-C-TG, RIBEYE-C-TG transgene.
