# Supplementary material for: The Making of Synaptic Ribbons: Carboxy-Terminal Tagging of RIBEYE B-Domain Reduces Steady-State Levels of RIBEYE and Its Efficacy to Build Synaptic Ribbons
Source: Cells. 2026 Aug 31;15(17):1583. doi: 10.3390/cells15171583 (PMC13565266; doi:10.3390/cells15171583)
Supplement: Supplementary file 1 [file cells-15-01583-s001.zip › cells-4480312-supplementary.pdf]

Supplementary Materials

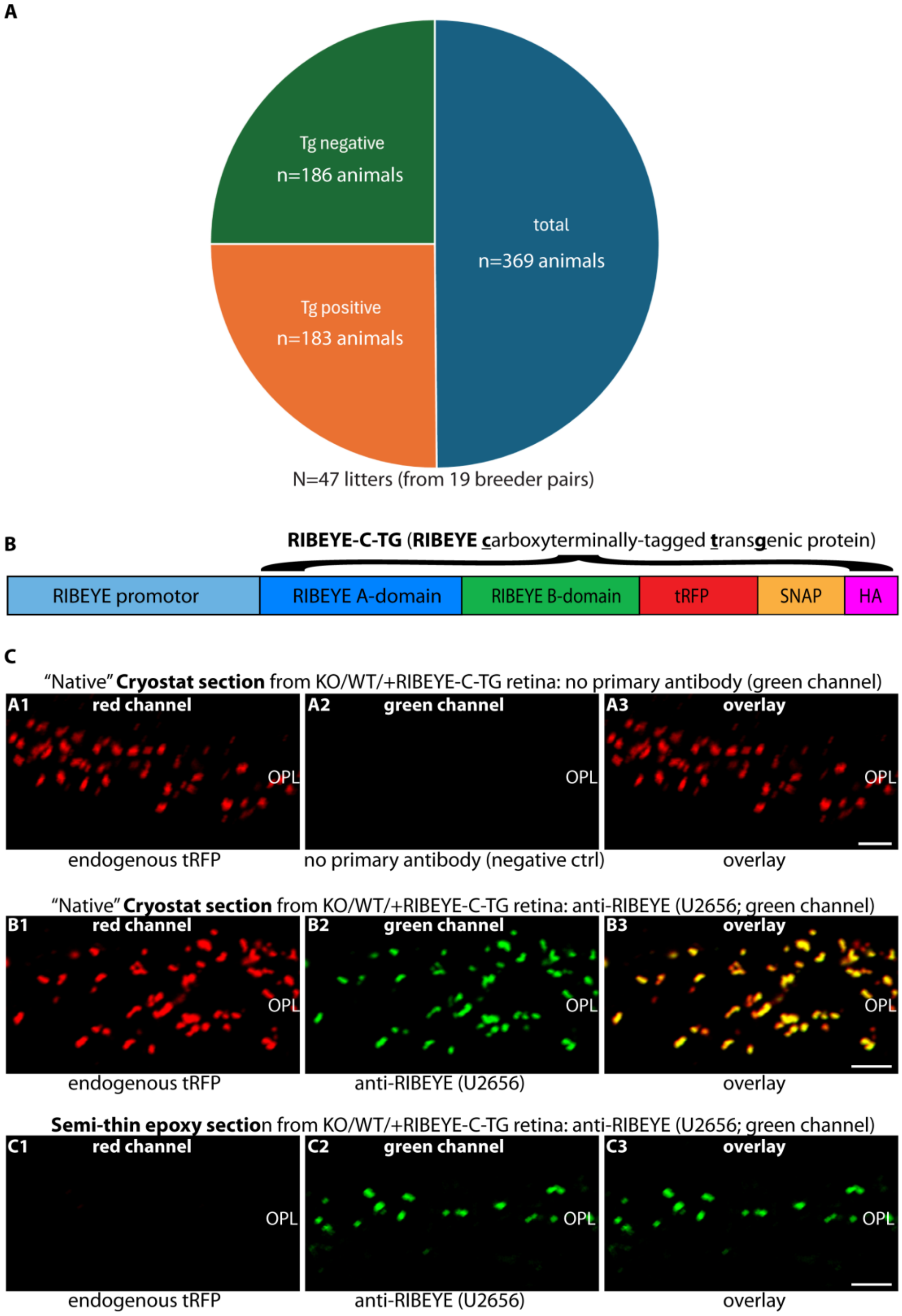

Supplemental Figure S1. (A) **A single copy of the transgene is present in the RIBEYE-C-TG transgenic mouse line.** The mouse offspring generated from breedings between a transgenically positive parent animal and a non-transgenic parent animal for the presence of the RIBEYE-C-TG transgene in the respective offspring. 47 litters from 19 breeder pairs were analyzed. Nearly exactly 50% of offspring from these litters were transgene-positive demonstrating that a single transgenic copy is present in the transgenic animals. (B) **Schematic depiction of the transgene encoding**

RIBEYE with the indicated protein tag fused to the carboxy-terminus of RIBEYE B-domain. C) **In contrast to native cryostat sections, the tRFP protein tag of RIBEYE-C-TG is not fluorescent in semi-thin epoxy resin sections**. Native cryostat sections (A,B) and semi-thin epoxy resin sections (C, after resin removal) from the indicated RIBEYE-C-TG-positive mice were analyzed for tRFP fluorescence in the red channel (excitation with the 561nm laser line; A1,B1,C1) and for RIBEYE immunosignals, obtained with rabbit polyclonal antibody against RIBEYE B-domain (U2656) and donkey anti-rabbit Alexa488 secondary antibody in the green channel (excitation with the 488 nm laser line; A2,B2,C2). Signals from the individual channels were overlaid in (A3,B3,C3). The endogenous fluorescence of the tRFP protein tag of the RIBEYE-C-TG is lost in the resin-embedded epoxy resin sections (C1). Abbreviations: Tg negative, transgene negative; Tg positive, transgene positive; RIBEYE-C-TG, RIBEYE-carboxy-terminally tagged transgenic protein; negative ctrl, negative control.

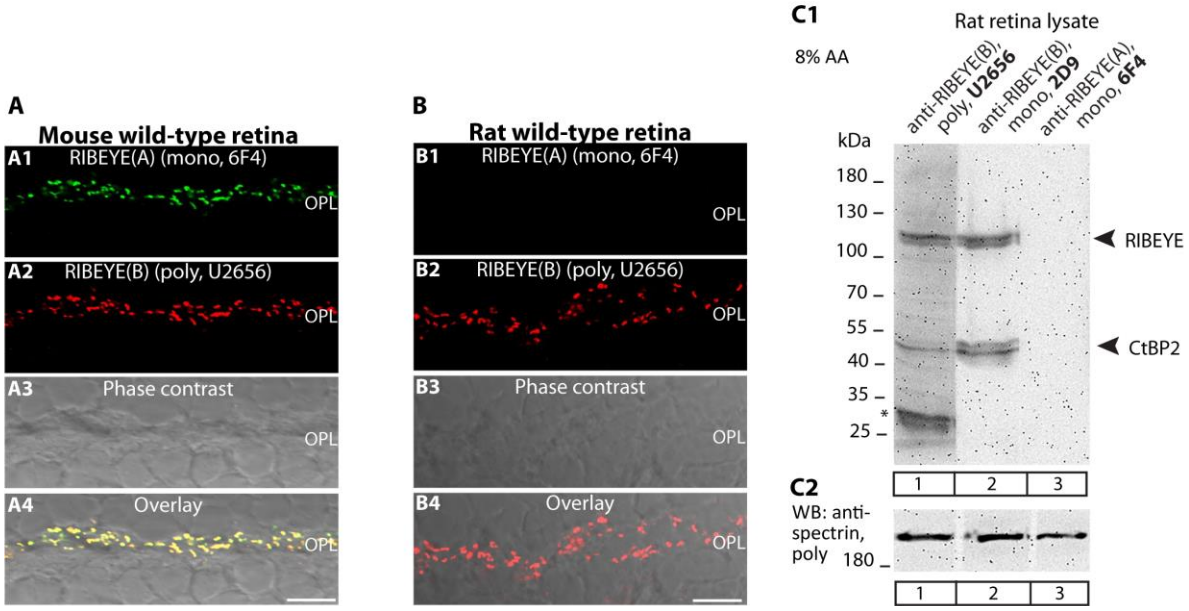

Supplemental Figure S2. The monoclonal antibody against RIBEYE-A-domain (6F4) detects RIBEYE in a species-specific manner. (A,B) The monoclonal RIBEYE A-domain antibody 6F4 detects RIBEYE in the mouse retina (A1), but not in the rat retina (B1), as shown by immunolabeling of rat and mouse retina cryostat sections (A,B). The rabbit polyclonal antibody against RIBEYE B-domain (U2656) detects synaptic ribbons in the OPL of the mouse retina (A2) and in the OPL of the rat retina (B2) demonstrating that synaptic ribbons were present in both species (positive control). (A3) and (B3) are phase contrast images of the respective sections. In (A4) and (B4) all respective single channels were merged. (C) WB experiments also confirmed that the 6F4 antibody does not detect rat RIBEYE in lysates of the rat retina (lane 3, C1). Rat RIBEYE is clearly present in the rat retinal lysate as demonstrated with the mouse monoclonal antibody against RIBEYE B-domain (clone 2D9, lane 2, C1) and rabbit polyclonal antibody against RIBEYE B-domain (U2656; lane 1, C1). In C2, the same lanes as shown in C1 were incubated with rabbit polyclonal antibody against brain spectrin. Brain spectrin served as loading control. The WB was analyzed with a BioRad ChemiDoc XRS system and with ECL detection. The asterisks (*) marks a degradation product of RIBEYE/CtBP2 that is detected by the U2656 antibody. Abbreviations: AA, acrylamide; OPL, outer plexiform layer; WB, Western blot. Scale bars: 5 µm.

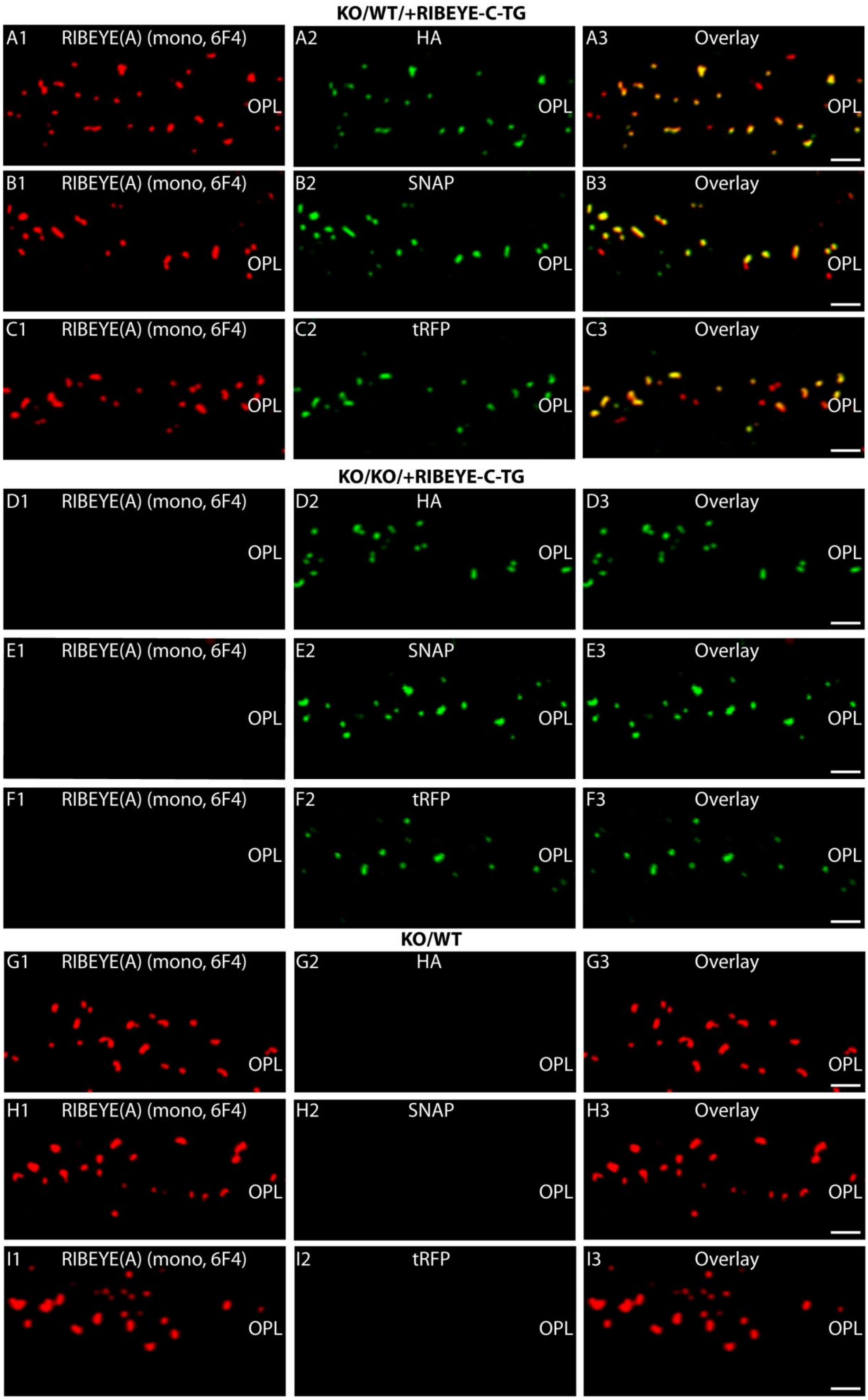
KO/WT/+RIBEYE-C-TG
A1 RIBEYE(A) (mono, 6F4)
A2 HA
A3 Overlay
B1 RIBEYE(A) (mono, 6F4)
B2 SNAP
B3 Overlay
C1 RIBEYE(A) (mono, 6F4)
C2 tRFP
C3 Overlay
KO/KO/+RIBEYE-C-TG
D1 RIBEYE(A) (mono, 6F4)
D2 HA
D3 Overlay
E1 RIBEYE(A) (mono, 6F4)
E2 SNAP
E3 Overlay
F1 RIBEYE(A) (mono, 6F4)
F2 tRFP
F3 Overlay
KO/WT
G1 RIBEYE(A) (mono, 6F4)
G2 HA
G3 Overlay
H1 RIBEYE(A) (mono, 6F4)
H2 SNAP
H3 Overlay
I1 RIBEYE(A) (mono, 6F4)
I2 tRFP
I3 Overlay
OPL

Supplemental Figure S3. Transgenic RIBEYE-C-TG is incorporated into endogenous synaptic ribbons in heterozygous RIBEYE (KO/WT) mice containing a single WT allele. Representative image of semi-thin retina sections of KO/WT/+RIBEYE-C-TG mice (A,B,C), KO/KO/+RIBEYE-C-TG mice (D,E,F) and KO/WT mice (G,H,I), double-immunolabeled with rabbit polyclonal antibodies against the HA-tag (A2,D2,G2)/SNAP-tag (B2,E2,H2)/tRFP (C2,F2,I2) and mouse monoclonal antibody against RIBEYE A-domain (clone 6F4) (A1,B1,C1,D1,E1,F1,G1,H1,I1). Signals from the red and green channels were overlaid in the right figure panels (A3,B3,C3,D3,E3,F3,G3,H3,I3). In KO/KO/+RIBEYE-C-TG mice, no immunosignal was observed with the RIBEYE A-domain antibody 6F4 (D1,E1,F1) because the 6F4 monoclonal antibody is specific for mouse RIBEYE and does not detect transgenic rat RIBEYE (see Suppl. Fig. 2). In KO/WT mice, no immunosignal was observed with the antibodies against the HA-tag (G2), SNAP-tag (H2) and tRFP-tag (I2) because no carboxy-terminal tagged RIBEYE-C-TG is present in these mice demonstrating the specificity of the immunolabeling data obtained with the tag antibodies. Immunosignals generated by the rabbit polyclonal antibody against the HA-tag (A2)/SNAP-tag (B2)/tRFP (C2) and by the mouse specific monoclonal RIBEYE antibody clone 6F4 (A1,B1,C1) show a high degree of colocalization in KO/WT/+RIBEYE-C-TG mice. (A1-A3: Manders' coefficient 1 (green signals overlapping with red signals, M1): 0.8080; Manders' coefficient 2 (red signals overlapping with green signals, M2): 0.8508 (N=4; n=52); B1-B3: Manders' coefficient 1 (green signals overlapping with red signals, M1): 0.9251; Manders' coefficient 2 (red signals overlapping with green signals, M2): 0.7841 (N=4; n=46); C1-C3: Manders' coefficient 1 (green signals overlapping with red signals, M1): 0.8232; Manders' coefficient 2 (red signals overlapping with green signals, M2): 0.7736 (N=4; n=50). Abbreviations: OPL, outer plexiform layer; KO, knockout; WT, wild-type; RIBEYE-C-TG, RIBEYE-C-TG transgene; N, number of mice; n, number of analyzed images. Scale bars: 5 µm.

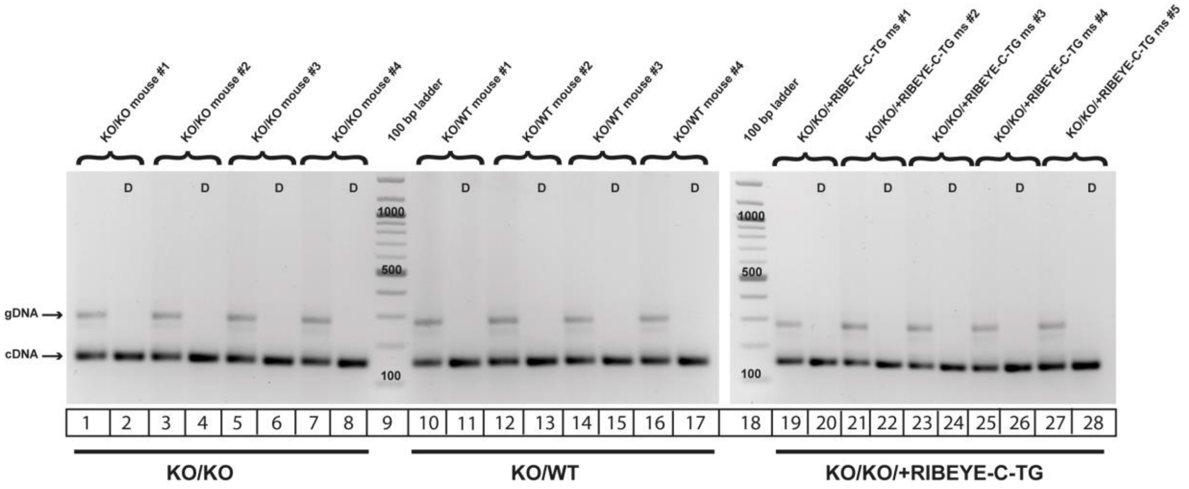

Supplemental Figure S4. Genomic DNA is efficiently removed by DNase digestion. Documentation of removal of genomic DNA of the indicated samples by using primers located in different exons of the actin gene separated by an intron. For this purpose, we used the β-actin (Actb)-specific probe-based HEX dye-conjugated assay (MmPT39a.22214843g, Integrated DNA Technologies, Coralville, IO, USA) to discriminate between cDNA and genomic DNA. If a genomic DNA contamination is present, an amplicon of 272 bp is observed which includes the 125 nucleotide long spanning intron; if only cDNA is present an amplicon of 125 bp is generated. After treatment with DNase (D; in lanes 2,4,6,8,11,13,15,17,20,22,24,26,28), the 272 bp PCR band indicating contaminating genomic DNA was completely absent in all samples indicating efficient removal of genomic DNA by the applied DNase-treatment. Lanes 1,3,5,7,10,12,14,16,19,21,23,25,27) show the same samples but before treatment with DNase. In lanes 9 and 18 DNA size standards are shown. Abbreviations: D; DNase-treated sample; gDNA, genomic DNA; cDNA, copy DNA; HEX,

hexachlorfluoresceine; KO, knockout; WT, wild-type; RIBEYE-C-TG, RIBEYE-C-TG transgene.
